# A Novel Intravital Imaging Window for Longitudinal Microscopy of the Mouse Ovary

**DOI:** 10.1038/srep12446

**Published:** 2015-07-24

**Authors:** Filip Bochner, Liat Fellus-Alyagor, Vyacheslav Kalchenko, Shiri Shinar, Michal Neeman

**Affiliations:** 1 Department of Biological Regulation, The Weizmann Institute of Science, Rehovot 76100 Israel; 2 Department of Veterinary Resources, Weizmann Institute, Rehovot 76100 Israel

## Abstract

The ovary is a dynamic organ that undergoes dramatic remodeling throughout the ovulatory cycle. Maturation of the ovarian follicle, release of the oocyte in the course of ovulation as well as formation and degradation of corpus luteum involve tightly controlled remodeling of the extracellular matrix and vasculature. Ovarian tumors, regardless of their tissue of origin, dynamically interact with the ovarian microenvironment. Their activity in the tissue encompasses recruitment of host stroma and immune cells, attachment of tumor cells to mesothelial layer, degradation of the extracellular matrix and tumor cell migration. High-resolution dynamic imaging of such processes is particularly challenging for internal organs. The implementation of a novel imaging window as reported here enabled longitudinal microscopy of ovarian physiology and orthotopic tumor invasion.

Preclinical studies on ovarian function and pathology require application of imaging approaches that would give mechanistic insight into dynamic biological processes occurring in the ovary. Noninvasive imaging of internal organs can be achieved with whole-body imaging techniques such as MRI. This approach, however, does not provide insight into cell migration, angiogenesis, and extracellular matrix remodeling at the cellular level. Complementary intravital multiphoton microscopy can be used for high-resolution imaging, albeit at limited penetration depth, requiring the use of transparent window preparations^1^.

Thus far, the optical imaging of the ovary has been mostly used as a tool for *ex-vivo* detection and quantification of changes associated with cancer progression. Several studies explored a possibility of using multiphoton excited autofluorescence, optical frequency multiplication as well as fluorescence lifetime imaging, to characterize tumor sections from patients^2^,^3^,^4^, and experimental animals^5^,^6^. Intravital multiphoton microscopy of the ovary with a stick-objective lens allowed visualizing neoplastic and normal ovarian surface epithelium^7^. Although these methods are promising for development of novel diagnostic strategies applicable in clinics, they do not enable longitudinal imaging studies on the ovary in preclinical setting.

Intravital imaging windows were successfully used to perform longitudinal observations, without a need for repeated surgeries. Thus far, several imaging windows designed to facilitate optical access to internal organs in mice, were developed. The most popular preparation is the dorsal skinfold chamber, which was used for longitudinal studies on hypoxia and vascular function^8^ as well as ECM remodeling in tumors^9^. Mammary imaging window was utilized to study orthotopic tumor microenvironments^10^. Development of abdominal imaging window enabled imaging of pre-micrometastasis in the liver^11^. Repeated imaging of vessel morphology, dendritic cells and microglia as well as studies on brain electrophysiology were performed in the cranial window^12^. Implementation of spinal cord imaging window enabled repetitive imaging of axon regeneration^13^. In this study, we demonstrate the development of an imaging window for *in-vivo* longitudinal microscopy of the murine ovary.

To examine ovarian structure in high resolution with two-photon microscope, endogenous autofluorescence of NADH, second harmonic signal of collagen and fluorescently labeled contrast agents were utilized to visualize cellular compartment, extracellular matrix, lymphatic network and the vasculature both *ex-vivo* in fresh, unsectioned tissues and *in-vivo* in the imaging window. It was feasible to image the full ovarian cycle, including follicular growth, and expansion of blood vessels *in-vivo*. Finally, upon xenotransplantation of fluorescent reporter expressing ES-2 cells into the nude mouse, orthotopic tumor formation was monitored over time. Recruitment of host’s cells to the tumor, formation of invasive tumor stroma, attachment of tumor cells to mesothelial layer and basement membrane as well as tumor spread on collagen fibers were imaged *in-vivo*.

## Results

### The ovarian imaging window

**Figure 1:**
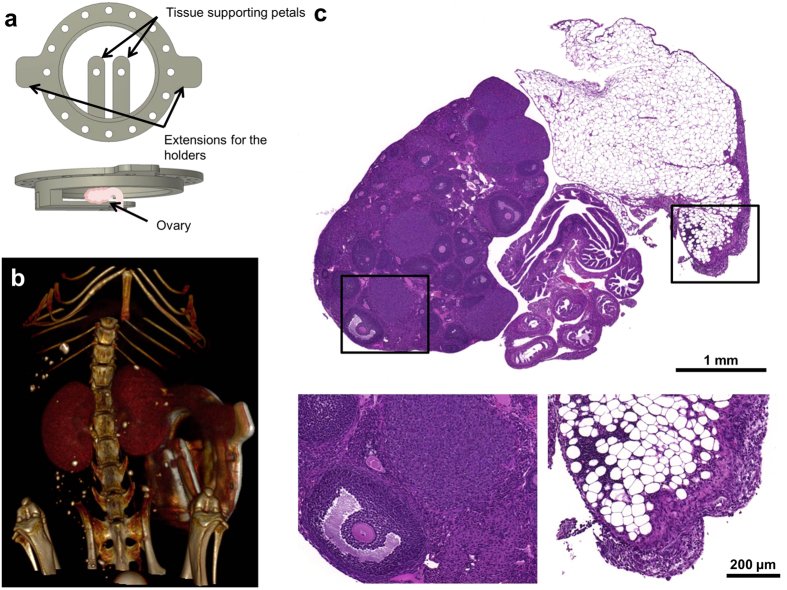
Scheme and localization of the imaging window on the mouse; tissue morphology after chamber implantation. (**a**) Schematic representation of the ovarian imaging window. (**b**) Post-contrast microCT image of the mouse bearing the ovarian imaging window. (**c**) Hematoxylin and eosin-stained histological section of the ovary kept in the imaging window for 7 days. Magnified regions denote antral follicle next to the corpus luteum, and mildly inflamed fat pad. **b** was imaged *in-vivo*.

The ovarian imaging window was constructed in-house from pure grade titanium. A titanium ring was shaped to provide a support for the ovary which after exteriorization from abdominal cavity, was positioned between titanium petals and a cover slip held in place with a plastic c-clip (Fig. 1a). The imaging window was mounted on the right dorso-lateral side of the mouse (Fig. 1b). First, a circular incision was made in the skin, exposing the peritoneal membrane below. The imaging window was stitched into the margins of the round skin cut. Upon making a small incision in the peritoneum, the ovary together with the oviduct were pulled outside the body cavity. Then, cyanoacrylate was used to glue the ovary by the surrounding fat pad into the tissue-supporting petals of the imaging window. Afterwards, the mouse was transferred to the custom-made imaging table and positioned under the stereomicroscope. The ovarian bursa was carefully removed to assure better visibility of the ovary and the oviduct. Subsequently, a sterile coverslip with a thin layer of hyaluronic acid spread on top was gently pressed against the ovary, and stabilized by a custom-made plastic c-clip. Finally, some cyanoacrylate was added to prevent opening of the sutures. The custom-made table and the imaging window were designed to minimize motion during imaging. The ovary was stabilized inside the imaging chamber with the tissue supporting petals and the coverslip. The entire imaging window was gently pulled up from the body and firmly attached to the imaging table with the clamps, which helped to isolate the ovary from the breathing motion of the abdomen (Supplementary Fig. S1 and Supplementary Video S1). Technical drawings for manufacturing the entire setup are available in Supplementary Materials. The ovaries showed no damage after 7 days inside the imaging chamber. Signs of inflammation were observed in the surrounding fat pad in all control animals. Inflammation was graded as mild to moderate. It involved some macrophage and neutrophils infiltration as well as mild fibrosis. The changes were exclusive to the fat pad, and did not affect the ovaries (Fig. 1c).

### Visualizing the ovarian morphology

**Figure 2:**
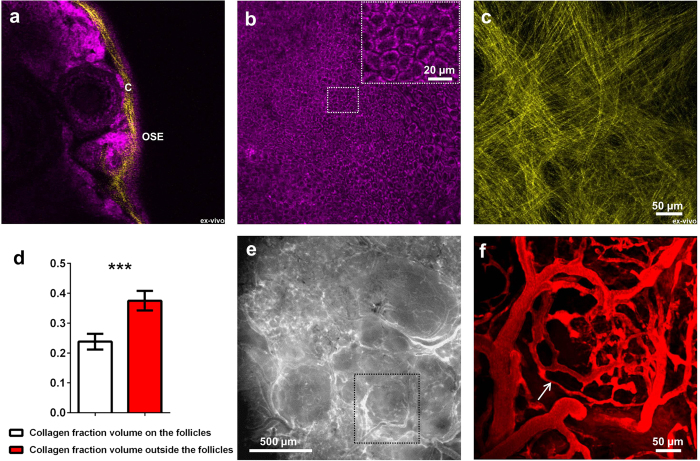
Imaging of the ovarian structure, ovarian surface epithelium, underlying basement membrane, localization and structure of the ovarian collagen, and vasculature. (**a**) Optical section through the edge of the ovary. (**b**) Optical section through ovarian surface epithelium, central region magnified in upper-right corner. (**c**) Projection of collagen underlying epithelial layer. (**d**) Difference in collagen density between the regions located on top, and outside the ovarian follicles, expressed as a collagen fraction volume. Fraction volume was averaged for 3 ROIs per group, per z-stack (n = 5, different animals). Unpaired, two-tailed student t-test revealed significant difference between the groups with ***P < 0.0001. (**e**) Stereomicroscope image of the ovarian vasculature upon injection of 500 kDa FITC – dextran. Region in the black frame was imaged also by two-photon microscopy (**f**). Blood vessel marked with the arrow is 3 μm in diameter. OSE – ovarian surface epithelium, C – collagen. Magenta – NADH, yellow – second harmonic generation, red – FITC-dextran. **a** and **c** were imaged *ex-vivo* in excised ovaries. **b**, **e** and **f** were imaged *in-vivo* in the imaging window.

**Figure 3:**
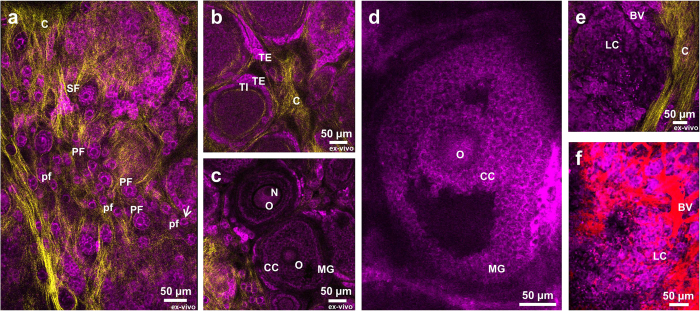
Stages of follicle maturation in the ovarian cortex. (**a**) Panoramic optical section of small ovarian follicles embedded in the collagen network. Primordial follicle marked with an arrow is 12 μm in diameter. (**b**) Optical section of late secondary follicles with separation on theca interna and theca externa. (**c**) Optical section through secondary follicles, with visible oocytes and separation between mural granulosa and cumulus cells (*ex-vivo*). (**d**) Optical section through growing antral follicle. (**e**) Optical section through corpus luteum. (**f**) Corpus luteum with visualized blood vessels upon injection with TRITC-dextran. pf – primordial follicle, PF – primary follicle, TE – theca externa, TI – theca interna, C – collagen, N – nucleus, O – oocyte, CC – cumulus cells, MG – mural granulosa, LC – luteal cells, BV – blood vessels. Magenta – NADH, yellow – second harmonic generation, red – TRITC-dextran. **a**, **b**, **c** and **e** were imaged *ex-vivo* in excised ovaries. **d** and **f** were imaged *in-vivo* in the imaging window.

As a basis for further investigation, the structure of ovarian cortex was examined with high detail. Autofluorescence of NADH was sufficient to image all developmental stages of the ovarian follicles both *in-vivo* in the imaging window and *ex-vivo* in freshly excised, unsectioned ovaries. Second harmonic generation signal was utilized to visualize collagen in the basement membranes. Optical cross-section through the edge of the ovary revealed orthogonal structure of collagen and arrangement of structures in the ovarian cortex (Fig. 2a). The externally located ovarian epithelium could be detected as a tight layer of cells with centrally located dark nuclei, and a bright ring of cytoplasm (Fig. 2b). This was consistent with the known cellular localization of NADH in the mitochondrial compartment and the cytoplasm. The network of collagen underlying ovarian surface epithelium consisted of a thin layer of linear, crisscrossing collagen fibers, outlining the margins of the follicular structures located below (Fig. 2c). Collagen layer located on top of the ovarian follicles was found to be thinner than collagen layer around them (Fig. 2d). Collagen fibers were also found inside the ovarian cortex, surrounding the ovarian follicles of different stages (Supplementary Fig. S2a). Collagen in the ovarian bursa had a structure resembling a spider web (Supplementary Fig. S2b), or alternatively appeared as parallel strands with a distinct helical morphology (Supplementary Fig. S2c). Similarly, it was possible to image structure of the oviduct (Supplementary Fig. S3). The vasculature pattern, detected by intravenous administration of 500 kDa FITC – dextran, reflected the location of the ovarian follicles (Fig. 2e,f). Injection of rhodamine-labeled bovine serum albumin enabled visualization of lymphatic vessels (Supplementary. Fig. S4). All the stages of follicle development were observed. Primordial, primary and young secondary follicles embedded in the collagen network could be seen in 21 days old mouse (Fig. 3a). Mature preantral follicles with clear basement membrane separating granulosa and theca layers (Fig. 3b), early (Fig. 3c) and late (Fig. 3d) antral follicles with separation to mural and cumulus cells as well as corpora lutea (Fig. 3e,f) were imaged in sexually mature mice. Additionaly, thanks to endogenous tissue autofluorescence active remodeling of ovarian tissue could be captured on time-lapse videos (Supplementary Video S2).

### Imaging hormone-induced changes in the ovary

**Figure 4:**
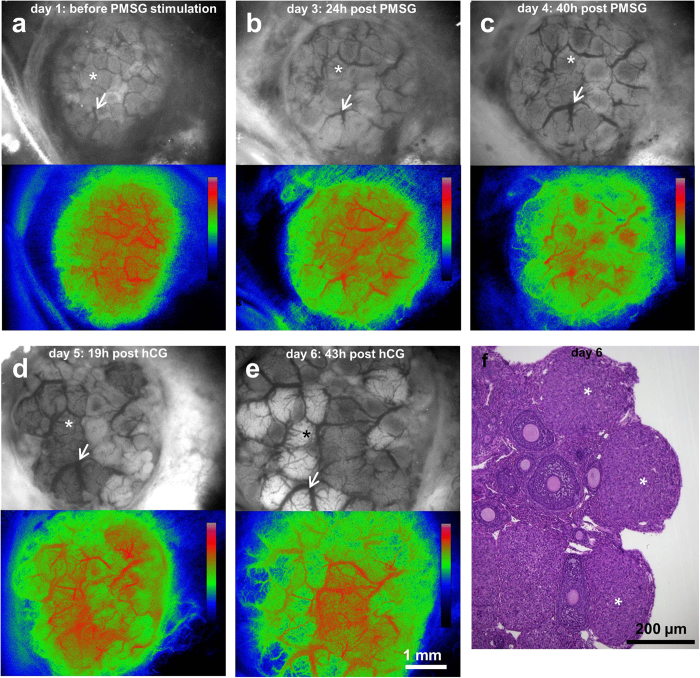
Physiological response of the ovary to hormonal stimulation. **(a–e)** Stereomicroscope images and perfusion maps of the mouse ovary undergoing hormonal changes upon injection of pregnant mare’s serum gonadotropin (PMSG; injected at day 2) and human chorionic gonadotropin (hCG; injected 48 h after PMSG, at day 4). Asterix indicates the same ovarian follicle at different time points; arrow denotes the same blood vessel. Calibration bars denote color-coded intensity range (0–255) which corresponds to blood flow map of each time point. **(f)** Hematoxylin and eosin-stained histological section of the ovary showing corpora lutei formed after ovulation. Asterix denote corpora lutea. **a-e** were imaged *in-vivo* in the imaging window.

The imaging window was mounted in animals on day 0, and imaged with stereomicroscope 24 h after, on day 1 (baseline) (Fig. 4a). At this stage, the mice were sexually immature, therefore no endogenous gonadotropins were present. On day 2, they were treated with an FSH analogue, pregnant mare’s serum gonadotropin (PMSG; subcutaneous). 22 h–24 h after, on day 3, induction of follicle growth and major vascular remodeling was observed (Fig. 4b). This process continued throughout day 4, 39 h–41 h after PMSG injection (Fig. 4c). These changes are characteristic for the FSH response. 48 h after PMSG stimulation, also on day 4, the mice were administered an LH analogue, human chorionic gonadotropin (hCG; intraperitoneal). 19 hours later, on day 5, further vascular remodeling, and change of color of some of the follicles were apparent, marking the formation of corpus luteum (Fig. 4d). On day 6, approximately 43 h after hCG injection a further increase of follicle size was observed (Fig. 4e). The incidence of multiple corpora lutea, as predicted after superovulation, was confirmed with histology (Fig. 4f). Vascular blood flow maps were acquired for each time point by speckle analysis. Blood circulation in the ovary was retained throughout the whole period of the experiment.

### Imaging the stages of tumor infiltration into the ovary

**Figure 5:**
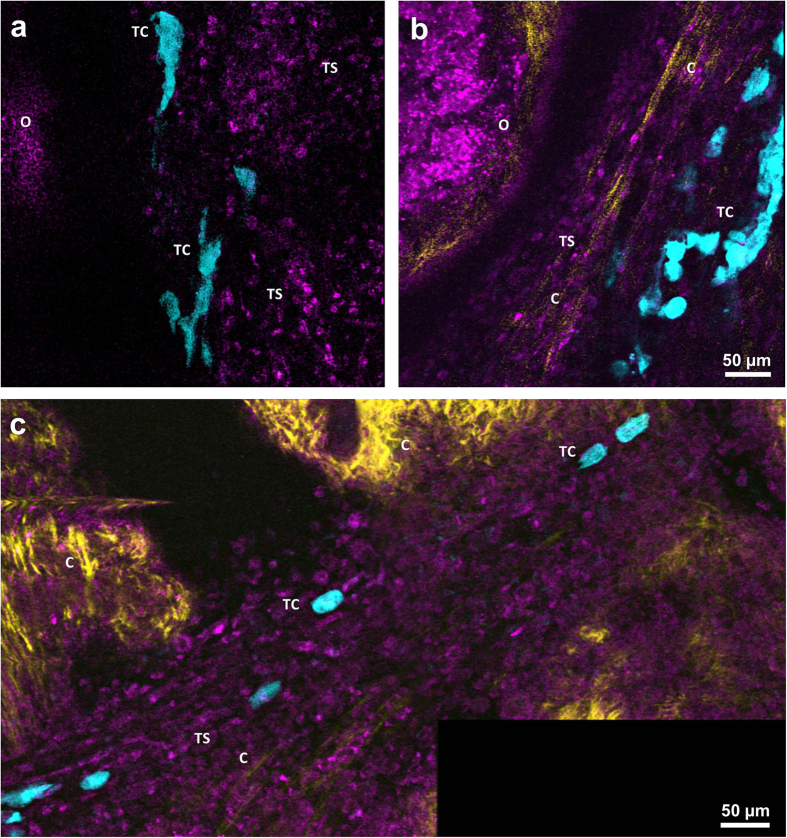
Formation of invasive tumor stroma. (**a**) Optical section through the tumor stroma surrounding the ovary 2 days after xenotransplantation. (**b**) Optical section through the tumor stroma at day 4 after xenotransplantation. (**c**) Optical section through the tumor stroma infiltrating the oviduct. O – ovary, TC – tumor cells, TS – tumor stroma, C – collagen. Magenta – NADH, yellow – second harmonic generation, cyan – tdTomato. All images were acquired *in-vivo* using the imaging window.

**Figure 6:**
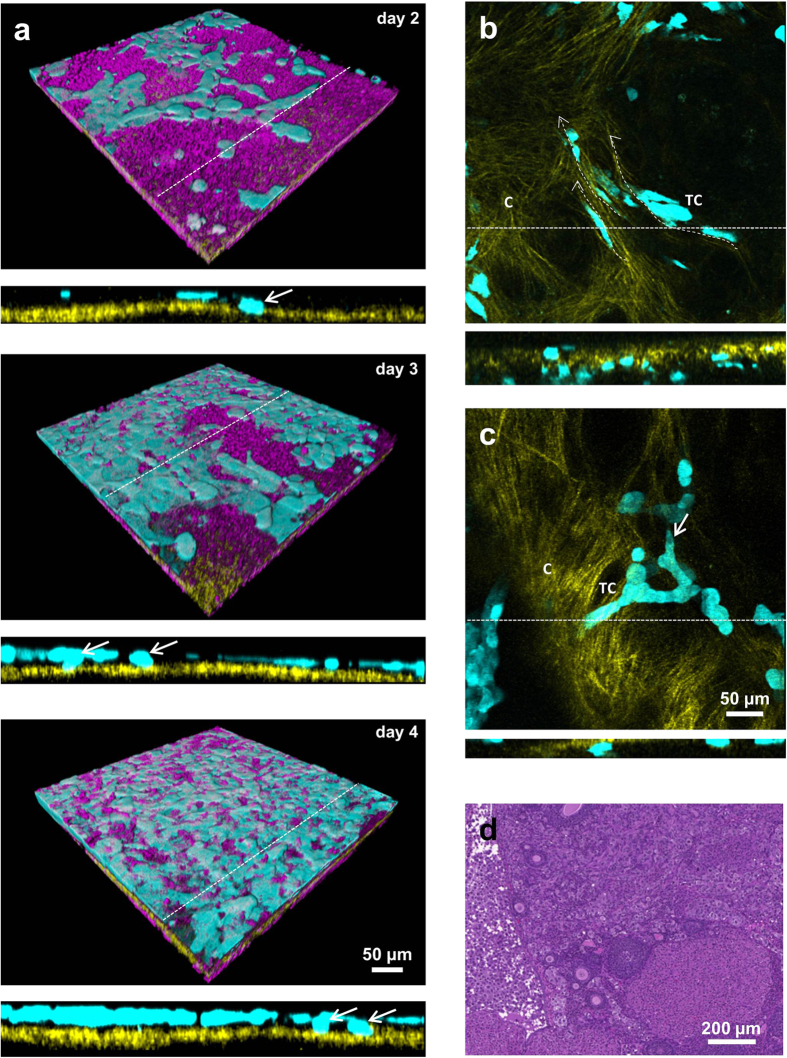
Stages of tumor infiltration into the ovary. (**a**) 3D projection of the same field of view imaged over period of 3 days (day 2–4 after xenotransplantation), showing spread of tumor cells above the mesothelial layer. Orthogonal sections (marked with dashed line) show the infiltration of tumor cells into the basement membrane underlying the mesothelial layer (not shown for clarity). (**b**) Optical section showing the tumor cells extended along the direction of the collagen fibers (dashed arrows). Corresponding orthogonal section (marked with dashed line) showing distribution of tumor cells across the basement membrane. (**c**) Maximum intensity projection showing tumor cells migrating as multicellular strand led by the tip cell (white arrow). Orthogonal view (dashed line) of the same field of view shows the attachment of the cellular strand into the basement membrane. (**d**) Hematoxilin – eosin stained histopathological section of the ovary infiltrated with the tumor cell, 14 days after xenotransplantation. C – collagen, TC – tumor cells. Magenta – NADH, yellow – second harmonic generation, cyan – eGFP. All images were acquired *in-vivo* using the imaging window.

To image tumor invasion into the ovary, tdTomato or eGFP – expressing human ovarian carcinoma ES-2 cells were xenotransplanted into the ovaries of the nude mice. Tumor cells in the vicinity of the ovary were found among the stromal cells (Fig. 5a) in parallel to collagen fibers (Fig. 5b). Similar stromal structures were located in the oviduct region (Fig. 5c). Elongated multicellular clusters could be seen extended over large distances (Supplementary Fig. S5a) Movement of cellular assemblies next to the ovarian edge appeared organized and directional in some areas and many tumor cells remained in contact with each other. Alternatively, single tumor cells were observed to invade the edge of the ovary (Supplementary Video S3). In the initial stage of breaching the mesothelial barrier, tumor cells attached to the ovarian surface epithelium and created a superficial monolayer. Only a small number of tumor cells detached from the monolayer and attached to the collagen underlying the ovarian surface epithelium. The process of tumor spread on the mesothelial surface and infiltration of the basement membrane was observed on three consecutive days on the same field of view (Fig. 6a). At laterstages, tumor cells could be found on both sides of the basement membrane. Cells on the internal side remained attached to the collagen and sent protrusion along the collagen fibers (Fig. 6b, Supplementary Video S4). They migrated as single cells or alternatively as “chains”, in which cells remained attached to each other (Fig. 6c). Localization of the tumor in the fat pad surrounding the ovary (Supplementary Fig. S5b) as well as inside the organ itself was confirmed by histolopathological analysis (Fig. 6d and Supplementary Fig. S5c). Tumor cells were also spotted on top of the oviduct collagen and inside its lumen (Supplementary Fig. S6) where their movement was possibly assisted by oviduct peristaltic motions (Supplementary Video S5).

## Discussion

Tissue morphogenesis in the ovary is dynamic and includes cyclic processes such as maturation of follicles, ovulation, and development and regression of corpora lutea. In addition, longitudinal changes during the female’s lifespan include the onset of puberty, continuous atresia, and loss of follicular reserve, leading to menopause and loss of ovarian function, as well as ovarian malignancies. Previous reports showed the possibility of utilizing endogenous autofluorescence and second harmonic signal to visualize the general structure of freshly excised, unsectioned and unstained murine ovaries^7^,^14^. In our work, the morphology of all the structures of the ovarian cortex was examined at detail together with the cellular dynamics followed *in-vivo*.

Ovarian surface epithelium plays an important role in ovarian physiology. It constitutes a dynamic layer of flat, tightly compacted cells, involved in transport of fluids, reduction of shear stresses, tissue repair and immune response^15^. Extracellular matrix in the ovary helps to maintain the tissue structure and integrity and regulates the steroidogenesis^16^. Degradation of the extracellular matrix was demonstrated to facilitate the follicular expansion^17^. Consistently, quantification of the collagen thickness on high-resolution z-stacks of the basement membrane showed thinning of the collagen on top of the growing ovarian follicles.

*Ex-vivo* two-photon imaging of autofluorescence and second harmonic signal in freshly isolated tissues constitutes an attractive approach to determine the structure of the unsectioned organ. The quality of *ex-vivo* and *in-vivo* images was comparable. Both types of images revealed similar features and details of the ovarian cortex and could be used to examine the ovarian structure. Based only on endogenous autofluorescence it was possible to discriminate between the cell layers surrounding the follicles of different stages, including differentiation between flat and cuboidal granulosa cells characteristic for primordial and primary follicles respectively, which are 12 μm in diameter. Although limited at depth, recognition and quantification of these structures by size and morphological features, using two-photon microscopy in freshly excised ovaries may constitute an alternative to the classic stereological methods of assessment of ovarian reserve performed on processed, histological sections^18^. Similarly, intravenous administration of fluorescent probe into the mice bearing ovarian imaging windows enabled *in-vivo* imaging of the blood vessels as thin as 3 μm in diameter, without a need of time-consuming tissue processing and 3D reconstruction of multiple histological images.

The ovary undergoes hormonally regulated changes manifested by growth of the ovarian follicles accompanied by the expansion of blood vessels. Whole body imaging techniques allow monitoring of tissue dynamics only at limited resolution. With the method employed here, the same ovarian follicles and blood vessels could be followed over time. Response of the ovary to hormonal stimulation and unimpaired perfusion of the blood vessels observed in the imaging window demonstrated viability of the method for the physiological studies.

The ovary is the origin and target organ of multiple malignancies, among which epithelial ovarian carcinoma is the most deadly among gynecological cancers. Tumor progression highly depends on its location. Clonal diversity of cancer cells is shaped by selective pressure of diverse microenvironments^19^, which includes involvement of multiple cell types that were demonstrated to facilitate tumor cell proliferation, migration and survival^20^. The ovarian imaging window can be utilized for monitoring orthotopic xenografts and their dynamic microenvironment over time in the nude mice. Possibly, it could also be used in genetic models of ovarian carcinoma.

Previous studies explored mechanisms of tumor invasion in *in-vitro* cultures or tissue sections from patients and animals. Observations made *in-vivo* in ovarian imaging window are consistent with the previous findings. In *in-vitro* mesothelial clearance assay ovarian cancer cells spheroids were shown to attach to the mesothelium and exert actin-myosin-driven force to relocate into the underlying basement membrane, while also clearing the mesothelium^21^. In ovarian imaging window, invading cancer cells formed a monolayer on top of the mesothelium; while some of the clones were found to intercalate below the mesothelial layer and make contact with underlying collagen. Cleavage of the basement membrane by the metalloproteases was shown to facilitate the attachment and invasion of the tumor cells to the underlying tissue^22^. Similarly, as demonstrated here, tumor cells initially injected to ovarian bursa, after breaching the mesothelium and underlying basement membrane, were eventually found inside the ovary, which was possibly associated with the action of metalloproteases. Tumor cells were shown before to use preexisting and modified collagen fibers as a substrate for migration^23^. Likewise, because of clonal and microenvironmental diversity, *in-vivo* imaging in ovarian window demonstrated that cancer cells utilize collagen fibers as migration tracks; while some of them remain stationary. Time-lapse microscopy confirmed that collective cell invasion is involved in tumor spread. Additionaly, cellular “chains” and assemblies previously described as multicellular invasion strands and detached clusters^24^, were captured on still images. Adipocytes were shown to attract tumor cells by secretion of cytokines and assure conducive microenvironment for their rapid growth by providing energy^25^. Congruently, as revealed by histopathological analysis, the majority of tumor mass was localized inside the adjacent fat pad. Most likely, shedding of tumor cells and growth of invasive tumor stroma towards the ovary, originated from tumor nodules formed inside that adjacent adipose tissue. The spread of cancer cells was also aided by peristaltic motions of the oviduct; they were passively moved from the ovary and peritoneal cavity towards other parts of reproductive system.

Formation and spread of ovarian cancer is aided by recruitment of mesothelial cells^26^, macrophages^27^ and fibroblasts^28^ that contribute to the formation of tumor stroma. Interestingly, although mesothelial cells lining the surface of peritoneal organs were shown to induce the tumor cell motility^26^, they also were able to protect the collagen matrices from tumor invasion by cell-to-cell contact^29^, which makes them an interesting and unexplored target for preclinical high-resolution imaging. Although recruitment of stromal cells to the tumor was observed thanks to endogenous autofluorescence and some could be identified by morphology, precise identification of the cellular subsets involved in invasion would require additional methodological approaches e.g. adoptive transfer of cells, co-transplantation of fluorescently labelled cell lines, or *in-vivo* labelling with antibodies.

The ovarian imaging window enabled high-resolution, longitudinal imaging of the orthotopic tumor formation, *in-vivo*. As we demonstrated here, infiltration of tumor cells through the mesothelial layer, collective and single-cell migration, recruitment of the cellular material of the host, formation of new collagen fibers, and invasive tumor stroma could be followed. Thus, in addition to providing a window for monitoring physiological ovarian function, during processes that affect female reproduction, the novel chamber reported here provides a significant tool for studying mechanism of progression in ovarian cancer, the most deadly gynecological malignancy, which is characterized by late detection and aggressive progression with very poor response to available therapeutic interventions.

## Methods

### Animal studies

Experiments were carried out in accordance with the Israel law on Experiments on Animals and Weizmann Institute approved guidelines. All experimental protocols were reviewed and approved by the Weizmann Institutional Animal Care and Use Committee.

Twenty-five days old, sexually immature C57/b mice (n = 4; Harlan, Rehovot, Israel) were used for imaging the ovarian cycle and the hormonal stimulation experiments. In the rest of cases, mice were 6–12 weeks old. CD1/nude mice (n = 12; Harlan, Rehovot, Israel) were used for the tumor xenotransplantation experiments. Imaging of ovarian microstructures was performed in C57/b mice (n = 11).

### Surgical procedure

The mice were anesthetized with Ketamine and Xylazine (i.p.; Ketamine 0.1 μl/g body weight; Xylazine 0.1 μl/g body weight) and placed on the feedback-controlled heating pad (FHC, USA). Since the left ovary is located adjacent to the spleen, the right ovary was chosen for surgical preparation to minimize the risk of bleeding. The skin on the right, dorso-lateral side of the mouse was disinfected with 70% ethanol solution. The skin at that site, just below the line of the ribs was gently pulled up with the forceps and slightly twisted to assure a circular shape of the incision. The resulting cut had approximately 12 mm in diameter. The intravital imaging window was positioned so that the two tissue-supporting petals were pointing in a direction parallel to the longitudinal axis of the mouse body. The imaging window was stitched to the edge of the incision. Silk thread (size 4-0 or 5-0) thread was pulled through the edge of the cut and one of the eyelets in the circumference of the window. It was optimal make a knot in each fifth of the eyelets and then continue with the remaining ones. Once the imaging window was stitched, another incision of approximately 2 mm was made in the peritoneal membrane. The ovary was exteriorized from the abdomen. To prevent a tissue damage caution was taken so the ovary manipulated by the surrounding fat pad. For immature mice, where the fat pad is not yet formed, the ovary was gently held by the uterine horn. LOCTITE acrylamide glue (Henkel, Israel) was applied on the tissue-supporting petals; the fat pad underlying the ovary together with the oviduct, and part of the uterine horn were gently pressed against them, and held until the glue solidified. Caution was taken so no glue leaked onto the ovary. It should be noted that for best optical access, the tissues should be placed in the center of the imaging window.

Next, the mouse was gently moved into the custom-made imaging table. Side-clamps were fixed and the mouse together with the imaging table was placed under the surgical stereomicroscope. For better optical access, the ovarian bursa was removed with a pair of sharp tweezers. After that, the imaging window was closed using a sterile coverslip with a layer of hyaluronic acid spread on the inner side, and sealed with an elastic plastic c-clip. Finally, the acrylamide glue was placed on all the stitching knots to prevent them from tearing. After surgery and in between the imaging sessions, the animals were kept in a heated recovery unit at 30 ˚C (Tecniplast, Buggagiate, Italy).

### Intravital imaging

Mice were anesthetized in an induction chamber with 3% isoflurane. Next, they were promptly transferred and mounted on a custom-built imaging table (see Supplementary Fig. S1). Plastic inserts supported the mouse from beneath, adjusted by the size of the animal. Throughout imaging sessions, isoflurane flow was maintained at 1.5%. The temperature was kept stable with feedback-controlled heating pad at 37.5 C. After temporarily disconnecting isoflurane outlet, the mice could be transported between microscopic modalities together with the imaging table. Isoflurane outlet was reconnected after approximately 30 s, which was sufficient to maintain continued anesthesia.

### Two-photon microscopy

Multiphoton microscopy was performed on LSM 510 Zeiss META NLO Laser Scanning Microscope with Plan Apochromat 20x/0.8 (Carl Zeiss, Germany), or Plan Apochromat 10x/0.45 lenses (Carl Zeiss); and Mai Tai HP DeepSee Ti:Sapphire femtosecond oscillator (Spectra Physics, Newport). Two-photon imaging was performed with 740 nm, 760 nm, or 880 nm. 390–465 nm, 435–485 nm, 500–550 and 575–615 nm band pass filters (Carl Zeiss, Germany) were used for the detection of second harmonic signal, NADH, eGFP/FITC – dextran, and tdTomato signal respectively. Alternatively, META detector (Carl Zeiss, Germany) was utilized to isolate the signal from the same emission sources and additionally from TRITC-dextran. The detection ranges set on the META detector for second harmonic signal, NADH, flavins and TRITC dextran were 373–383 nm, 437–458 nm, 512–533 nm and 608–619. No immersion was used. Specific image information is included in Supplementary Table S1.

### Stereomicroscopy

Imaging was performed on an Olympus SZX12 RFL2 microscope (Olympus, Japan) with DF PLAPO 1 x PF lenses (Olympus, Japan), coupled with PIXELFLY QE 12 bit CCD camera (PCO, Germany). U-RFL-T High Pressure Mercury Burner lamp (Olympus, Japan) was used as a source of excitation light and cold light reflector lamp KL-2500 LCD (Olympus, Japan) as a source of light for bright field illumination. Dynamic Laser Speckles Imaging was performed with with the ELFI-C laser illumination unit (Elfi-Tech Ltd., Israel). To obtain spectrally enhanced hemoglobin contrast images, white light was passed through a filter (excitation 460–490 nm, emission 510–550 nm; bandpass)(Croma, USA). For Dynamic Laser Speckles Imaging the illumination unit was placed approximately 10 cm from the tissue and 310 consecutive frames were acquired with the exposure of 65 ms. EGFP/FITC-dextran and tdTomato/BSA-rox, were imaged upon illuminating the tissue with excitation light from the illumination unit, and filtering the signal of fluorescent proteins with dichroic filters: DM-505 (excitation 460–490 nm, emission 510 –; longpass) and DM-580 (excitation 460–560 nm, emission 590 – ; longpass) respectively.

### CT

The ovarian imaging window-bearing mice were scanned in the TomoScope® 30S Duo micro-CT (CT imaging, Germany) following injection of Omnipaque (3 mg/g of body weight, through an intravenous catheter placed in the tail-vein). The protocol was performed with the tubes voltage of 40 kV; tube current of 450 μA; and exposure time of 90 ms. The mice were scanned immediately after Omnipaque administration.

### Superovulation induction in chamber bearing mice

Sexually immature C57/b mice bearing ovarian imaging window received subcutaneous injection of pregnant mare’s serum gonadotropin (PMSG; 5 IU, National Hormone & Peptide Program, Harbor-UCLA Medical Center, California, U.S.A), followed by intraperitoneal injection of human chorionic gonadotropin (hCG; 5 IU, Sigma Aldrich) 48 h later. Imaging was performed starting at 24 h after mounting the imaging window. The images were obtained on day 1 (baseline) and days 3–6. PMSG and hCG were injected at day 2 and 4 respectively. At each time point bright field image, enhanced hemoglobin contrast, and laser speckles maps of blood flow were calculated as previously reported.

### Visualization of blood and lymphatic vasculature

To visualize blood vessels 2 mg of 500 kDa FITC or TRITC-labeled dextran (SIGMA-Aldrich, Germany) was injected intravenously into in a tail vein through a catheter. The ovarian vasculature was imaged shortly after with stereomicroscope and two-photon microscope.

To visualize lymphatic vessels 2 mg of BSA (Sigma-Aldrich, Germany) labeled in-house with ROX (Molecular Probes) was injected intravenously into in a tail vein through a catheter (protocol modified from^30^). Significant decrease of the signal in blood vessels and leakage of BSA-rox to extracellular matrix was observed 30 min post-injection with stereomicroscope. At this point FITC-dextran was injected to provide the corresponding image of blood vasculature. Next, the mouse was sacrificed; the ovary was isolated and imaged with two-photon microscope.

### Histology

Hematoxylin and eosin staining was performed for histopathological evaluation of the tissues. After sacrificing a mouse, the imaging window was opened by removing a plastic c-clip and gently lifting the coverslip. In case of tumor experiments, the entire tissue including ovary, oviduct, fat pad and part of uterine horn was removed. Tissue was gently pulled up with a forceps, detached from the tissue-supporting petals of the imaging window and cut off with a scissors, approximately in the center of the uterine horn. As an endpoint of hormonal stimulation experiment, only the ovary was removed. In this case, the forceps were softly latched in between the ovary and the oviduct and pulled up to detach the ovary. Removed tissues were attached to small cardboards and fixated with 4% formaldehyde. 24 h later, they were transferred to 1% formaldehyde in DDW. Tissues were dehydrated and paraffinized with tissue processor TP 1050 (Leica, Germany), and embedded in paraffin blocks with EG 1160 (Leica, Germany). 4 μm-thick sections were taken from the ovarian cortex and the medulla. After placing the sections on the slides, they were dried and stained with an TST-40 automated stainer (Medite, Germany). After sealing with the coverslips the slides were imaged with the Panoramic MIDI automatic slide scanner (3D Histech, Hungary), or alternatively with Axio Observer.1 microscope (Carl Zeiss).

### Cell culture

Upon transfection with EGFP and tdTomato fluorescent proteins (backbone of pIRES vector, under the EF-1a promoter), ES-2 ovarian cancer cells (ATCC) were cultured in DMEM (Biological Industries, Israel) supplemented with 10% FCS and L-glutamine. Cells were harvested with 0.1% trypsin (Beit Haemek, Israel) and washed with the saline. 7 days before mounting the imaging window, approximately 3*10^6^ cells were injected into the ovarian bursa in 1% solution of hyaluronic acid (Biolon, Israel). Alternatively, during the imaging window mounting procedure, 3*10^6^ tumor cells in 1% hyaluronic acid was placed on the coverslip facing the ovarian tissue.

### Image processing

Blind 3D - deconvolution algorithm was applied to all two-photon images. Data was batch-processed with AutoQuant software (MediaCybernetics). Deconvolution was performed with 10 iterations, taking into account high noise levels. Optical settings were adjusted according to raw image specifications, taking into consideration emission spectra of Second Harmonic Signal (SHG), NADH, GFP and TRITC (see **two-photon microscopy**). Sample medium was assumed as water. For deconvolution in AutoQuant, images were split into separate channels and converted into 32 bit. Separated channels were merged back in ImageJ (open source) and saved as 16, or 8 bit. Brightness and contrast were adjusted if necessary. Images were not otherwise manipulated.

After deconvolution, time-lapses were recreated in ImageJ from subsequently acquired z-stacks. Bleach correction by histogram matching was applied to all movies. Time-lapses were further processed in Imaris (Bitplane, Switzerland). Brightness and contrast were adjusted if necessary. The movies were exported in tiff format, after recording the time-lapses in “Easy 3D” mode (maximum intensity projection with shading). The final movies were created in ImageJ after adding the annotations.

Images obtained with stereomicroscope were processed using ImageJ. Brightness and contrast was adjusted for spectrally enhanced images of hemoglobin and for fluorescent images. Laser Speckles 310 frames sequences containing were combined into single intensity map of the blood flow with custom-written ImageJ plugin^31^. The processing of the image into the intensity map was performed as described^31^. The resulting image was reciprocally inverted and calibration bar was embedded into the image. Resulting RGB image was converted into 16 bit. Gamma was adjusted if necessary. Rainbow RGB scale was chosen to represent the differences in blood flow within the single time points, in arbitrary intensity units. Specific image information is included in Supplementary Materials.

MicroCT image reconstruction and subsequent processing including artifact removal and surface rendering was performed in OsiriX (Pixmeo, Switzerland).

## Additional Information

**How to cite this article**: Bochner, F. *et al.* A Novel Intravital Imaging Window for Longitudinal Microscopy of the Mouse Ovary. *Sci. Rep.* **5**, 12446; doi: 10.1038/srep12446 (2015).

## Supplementary Material

Supplementary Information

Supplementary Video S1

Supplementary Video S2

Supplementary Video S3

Supplementary Video S4

Supplementary Video S5

Supplementary Information
