# Supplementary material for: A Novel Intravital Imaging Window for Longitudinal Microscopy of the Mouse Ovary
Source: Sci Rep. 2015 Jul 24;5:12446. doi: 10.1038/srep12446 (PMC4513547; doi:10.1038/srep12446)
Supplement: Supplementary Information [file srep12446-s1.pdf]

# A Novel Intravital Imaging Window for Longitudinal Microscopy of the Mouse Ovary

**Filip Bochner[1], Liat Fellus[1], Vyacheslav Kalchenko[2], Shiri Shinar[1] and Michal Neeman[1]**

[1] Department of Biological Regulation, and [2] Department of Veterinary Resources, Weizmann Institute, Rehovot 76100 Israel

**Supplementary material**

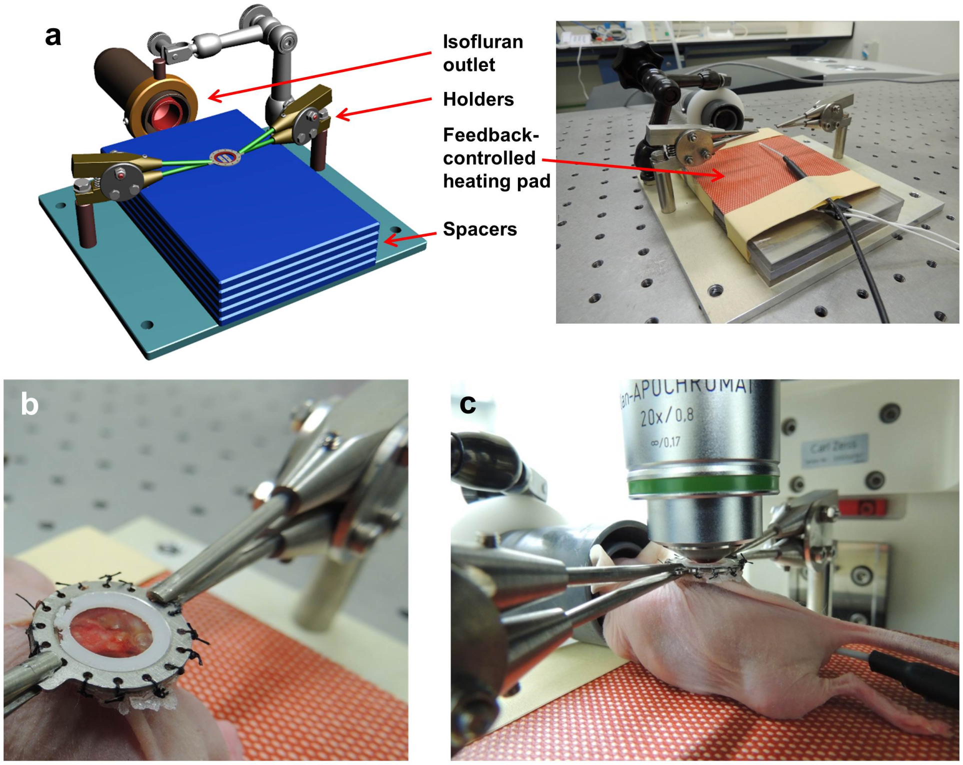


**Supplementary Figure S1** | Design of the imaging stage. (**a**) Schematic visualization of the system. After prompt disconnection from isoflurane anesthesia, the animal can be transferred between the optical imaging modalities together with the imaging stage (**b, c**). The imaging window is gently lifted from the body and stabilized with the holders. This helps to prevent breathing motions of the abdomen to be transmitted into the imaging window (see also **Supplementary Video 1**)

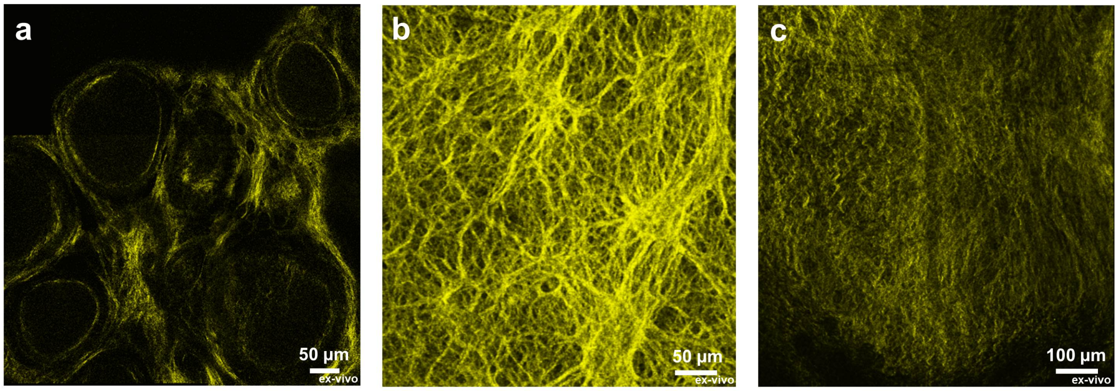


**Supplementary Figure S2** | (**a**) Panoramic optical section, localization of collagen in the ovarian cortex. (**b, c**) Projections of different collagen structures in the ovarian bursa. Yellow – second harmonic generation. All images were acquired *ex-vivo* from excised ovaries.

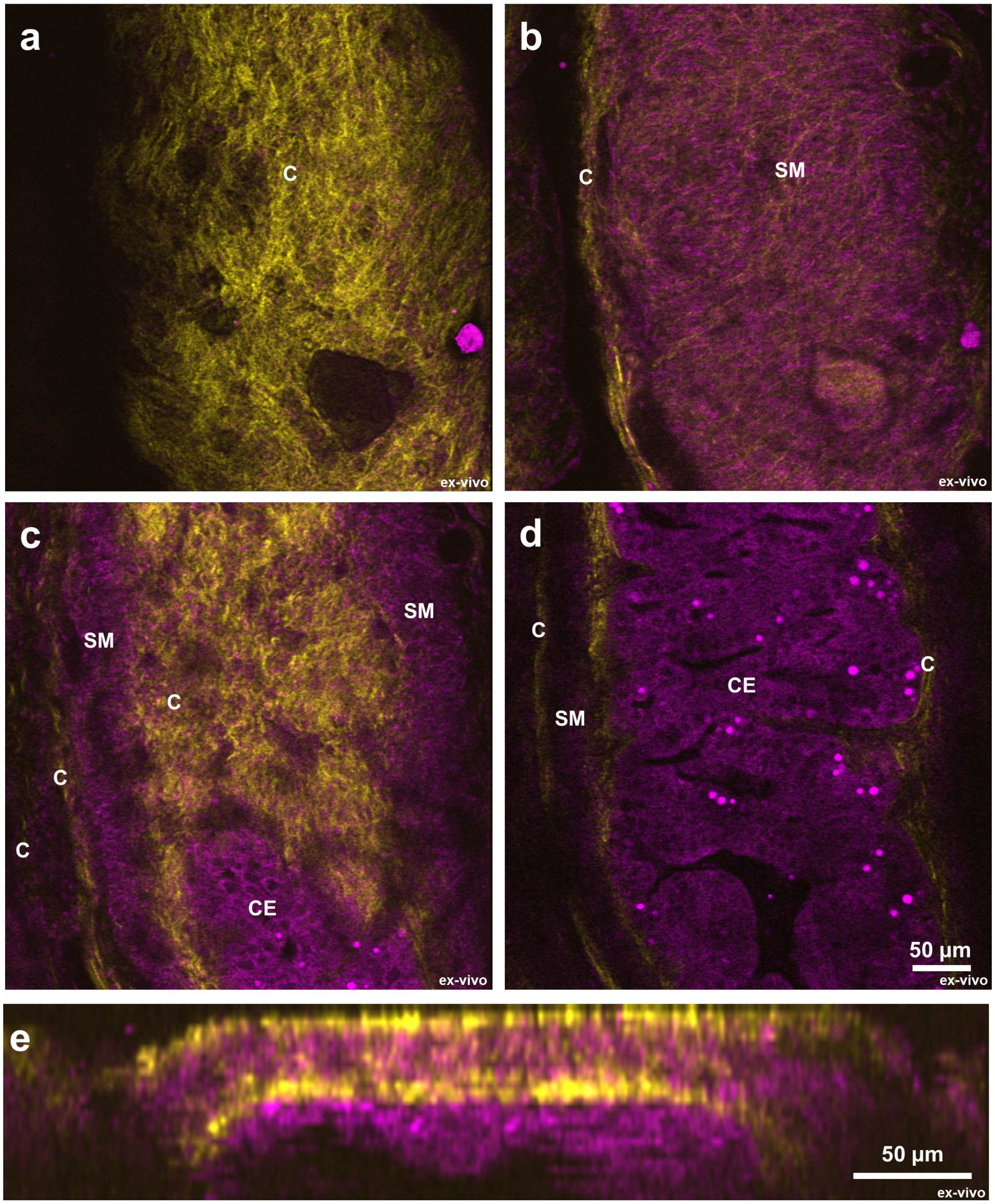


**Supplementary Figure S3** | Murine oviduct imaged at different depths. (**a**) 0 µm, (**b**) 15 µm, (**c**) 30 µm, (**d**) 54 µm, (**e**) orthogonal view of the oviduct. C – collagen, SM – smooth muscle, CE- columnar epithelium. Magenta – NADH, yellow – second harmonic generation. All images were taken *ex-vivo* in excised oviduct.

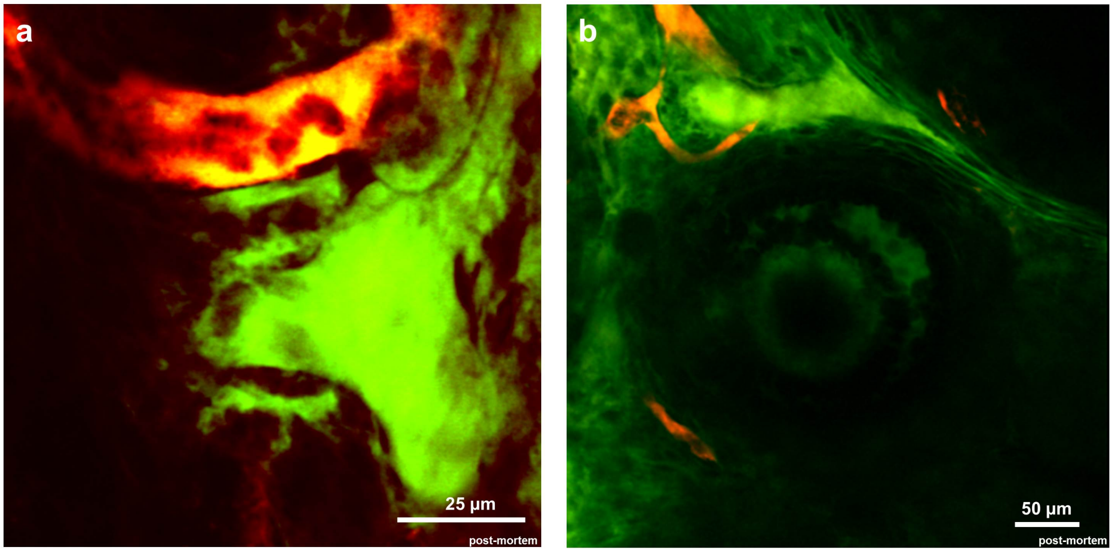


**Supplementary Figure S4** | Visualization of lymphatic vessels upon injection of rhodamine-labeled bovine serum albumin and fluorescein-labeled dextran. (**a**) Optical section through the lymphatic and blood vessel. (**b**) Optical section through the ovarian follicle. Green – BSA-ROX, orange – FITC dextran. All procedures were performed in the live animal, however these images were acquired immediately *post-mortem* using the imaging window.

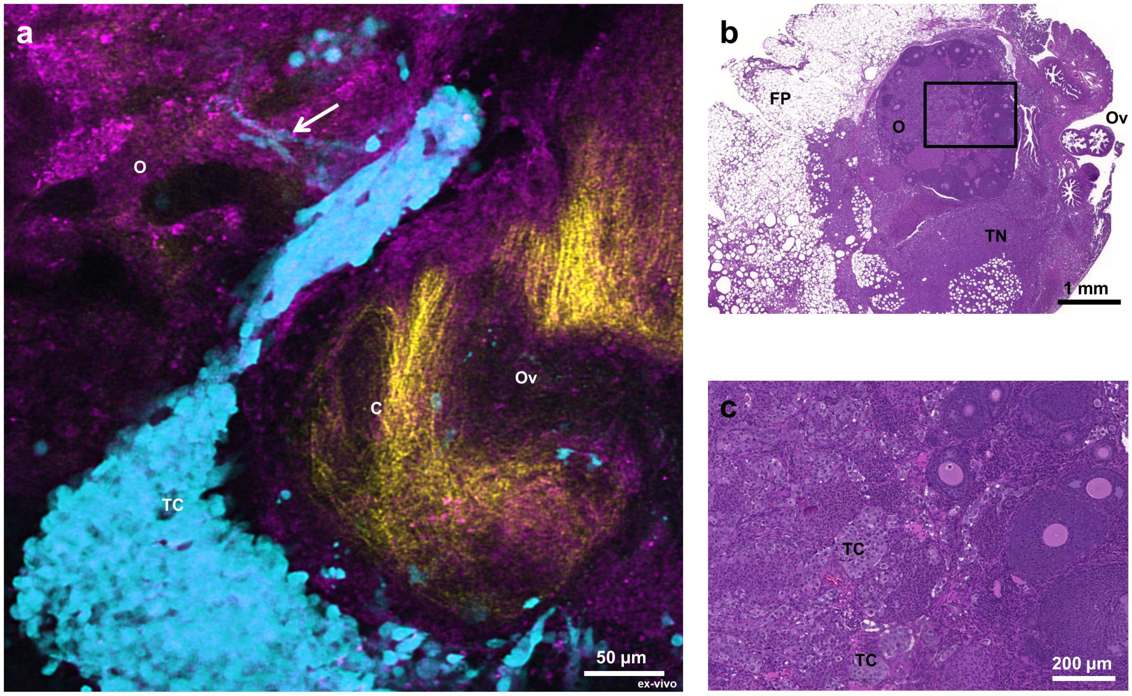


**Supplementary Figure S5** | (**a**) Two-photon optical section through multicellular cluster of tumor cells intercalating between the ovary and oviduct. Chains of cells extending from the main cluster marked with an arrow. (**b**) Hematoxilin – eosin stained histopathological section of the ovary region, infiltrated by tumor cells, 14 days after xenotransplantation. Upon injection into the ovarian bursa, tumor cells localized mainly into the fat pad surrounding the ovary spreading eventually spreading into the organ itself (region in the bracket is magnified in **c**). (**c**) Multicellular clusters of tumor cells inside the ovary. C – collagen, TC – tumor, O – ovary, Ov – oviduct, TN – tumor nodule, FP – fat pad. Magenta – NADH, yellow – second harmonic generation, cyan – eGFP. All the procedures were performed in the live animal, however image **a** was acquired *post-mortem* using the imaging window within a few minutes after death.

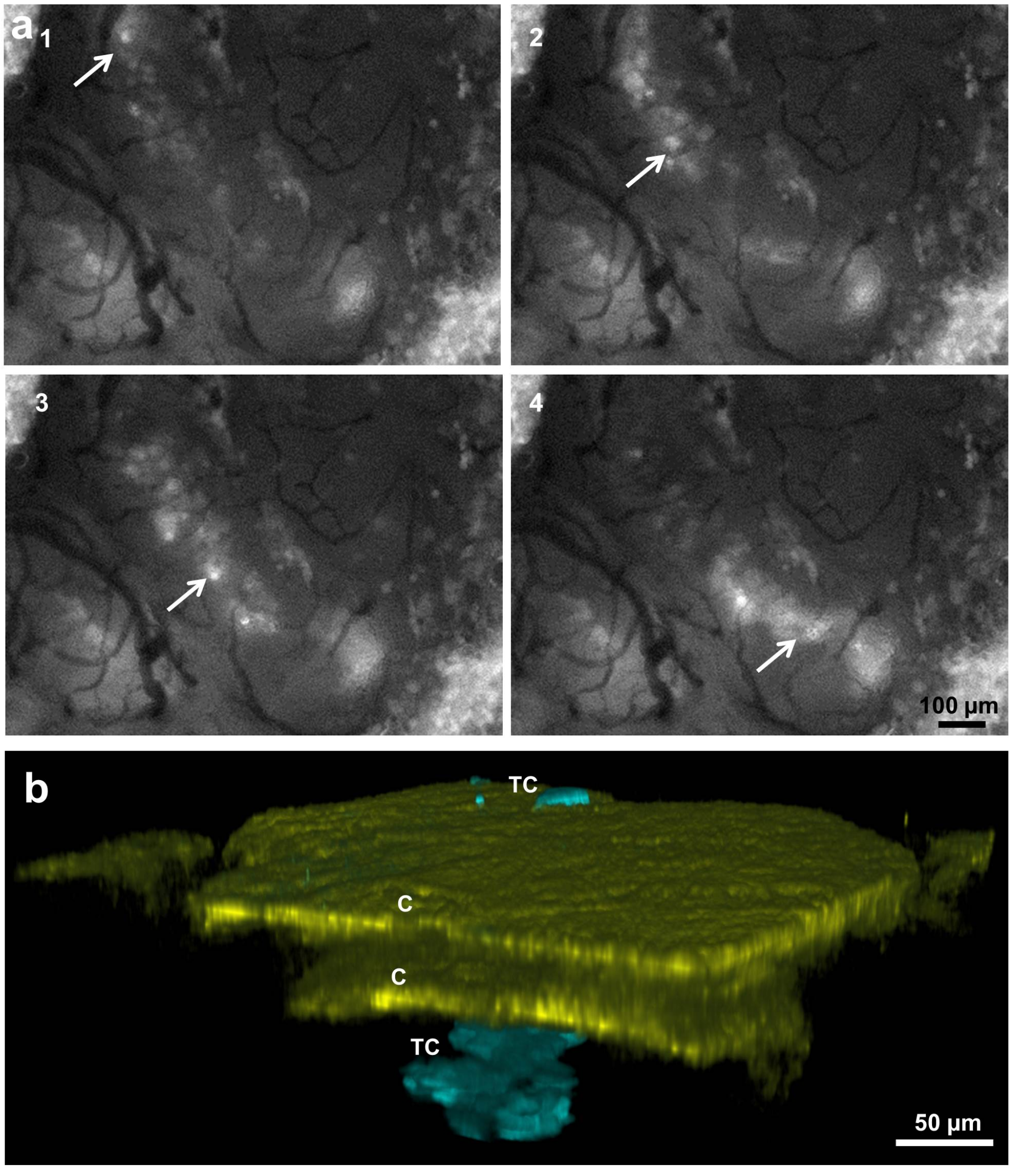


**Supplementary Figure S6** | Tumor cells spreading through the oviduct 12 days after xenotransplantation (**a**$_{1\text{-}4}$) Frames from the time-lapse video recorded with stereomicroscope, showing tumor cells being moved through the oviduct due to its contractile motions. White arrow denotes a group of tumor cells (see also **Supplementary Video 4**). (**b**) 3D-projection of two-photon z-stack showing tumor cells visible on the external layer of collagen and inside the lumen. TC – tumor cells, C – collagen. Yellow – second harmonic generation, cyan – eGFP. All images were acquired *in-vivo* in the imaging window.

**Supplementary Videos**

**Supplementary Video S1** | Principles of motion artifact elimination. The video shows one of the prototypes of the ovarian imaging window. The ovary attached to tissue-supporting petals is isolated from breathing motions. The breathing is not transmitted to the ovary, which is slightly pulled out from the body and while being attached to the imaging window, it is kept straight by the external holders. Coverslip (not seen on this video) placed on top of the tissue provides additional level of stabilization.

**Supplementary Video S2** | Dynamic remodeling of ovarian tissue. Thanks to autofluorescence of NADH (magenta) and FAD (green) it is possible to follow migration of cell populations that are endogenous to the ovary. Although it is impossible to determine their cell type, due to lack of specific labeling, they exhibit certain properties enabling their classification. For example presence of very active endocytic compartment in form of highly autofluorescent granules (green), size (~ 12 µm diameter) and amoeboid type of motion can be attributed to macrophages. The duration of acquisition of this video is 1 h 2 min.

**Supplementary Video S3** | Tumor invasion into the edge of the ovary. Some of the tumor cells (cyan) within the population remain in close contact with each other, and their movement appears directional. Some single tumor cells (small white arrows) cells move in the direction of the ovary and attach to its collagen (yellow). The edge of the ovary (here visualized thanks to SHG signal colored in yellow) is located in the top half of the video. Some of the tumor cells (cyan) are already attached to the collagen. The bottom part of the video contains mostly cancer cells in motion. As time passes, some of the cancer cells (marked with arrows) detach from the cluster outside of the organ, and “climb” on the ovarian collagen. Similar process is depicted in Supplementary Figure S5a, in which cancer cells are detached from the main cluster outside the ovary create a stream of cells invading the organ. The duration of recording of this video is 1 h 45 min.

**Supplementary Video S4** | Migration of tumor cells on collagen fibers. Tumor cells (cyan) utilize collagen fibers (yellow) as tracts for migration and sites of attachment. Some of the clones exhibit high motility (white arrows) sending cytoplasmic protrusions on the collagen, while others remain stationary. The duration of recording of this video is 3 h 36 min.

**Supplementary Video S5** | Spread of tumor cells through the oviduct. Tumor cells that spread in peritoneal cavity are being actively pumped by contractile movements of the oviduct towards more distant parts of the reproductive system. The black frame shows the area magnified in **Supplementary Figure S6a**

**Supplementary Table S1 | Specific two-photon image information**

| Figure | Stack resolution (0.82 μm/pixel) | Scan zoom | Objective lens/numerical aperture | Filters (nm) | Excitation wavelength (nm) | Z – increment (nm) |
|---|---|---|---|---|---|---|
| 2A | 0.82 | 1 | Plan-Apochromat 20x/0.8 | META:<br>373 – 383<br>437 – 458<br>512 – 533;<br>Band Pass: 575 – 615 IR | 760 | - |
| 2B | 0.21 | 1 | Plan-Apochromat 20x/0.8 | META:<br>373 – 383<br>437 – 458<br>512 – 533 | 760 | - |
| 2C | 0.41 | 1 | 20x/0.8 | META:<br>373 – 383<br>437 – 458<br>512 – 533 | 760 | 1.5 |
| 2F | 0.82 | 1 | 20x/0.8 | META:<br>373 – 383<br>437 – 458<br>512 – 533 | 760 | 2 |
| 3A | 0.41 | 1 | 20x/0.8 (stitched from two images) | META:<br>373 – 383<br>437 – 458<br>512 – 533 | 760 | - |
| 3B | 0.41 | 1 | 20x/0.8 | META:<br>373 – 383<br>437 – 458<br>512 – 533 | 760 | - |
| 3C | 0.41 | 1 | 20x/0.8 | META:<br>373 – 383<br>437 – 458<br>512 – 533 | 760 | - |
| 3D | 0.41 | 1 | 20x/0.8 | META:<br>373 – 383<br>437 – 458<br>512 – 533 | 760 | - |
| 3E | 0.82 | 1 | 20x/0.8 | META:<br>373 – 383<br>437 – 458<br>512 – 533 | 760 | - |
| 3F | 0.41 | 1 | 20x/0.8 | META: | 760 | - |

| | | | | | | |
|---|---|---|---|---|---|---|
| | | | | 373 – 383<br>437 – 458<br>608 – 619 | | |
| 5A | 0.82 | 1 | 20x/0.8 | META:<br>373 – 383<br>437 – 458<br>512 – 533;<br>(Band Pass:<br>575 – 615 IR) | 760,<br>(960) | - |
| 5B | 0.82 | 1 | 20x/0.8 | META:<br>373 – 383<br>437 – 458<br>512 – 533;<br>(Band Pass:<br>575 – 615 IR) | 760,<br>(960) | - |
| 5C | 0.82 | 1 | 20x/0.8<br>(stitched from 2 images) | META:<br>373 – 383<br>437 – 458<br>512 – 533;<br>(Band Pass:<br>575 – 615 IR) | 760,<br>(960) | - |
| 6A | 0.82 | 1 | 20x/0.8 | Band Pass:<br>435 – 485 IR<br>(390 – 465 IR<br>500 – 550 IR) | 740,<br>(880) | 2 |
| 6B | 0.82 | 1 | 20x/0.8 | 390 – 465 IR<br>500 – 550 IR | 880 | 2 |
| 6C | 0.82 | 1 | 20x/0.8 | 390 – 465 IR<br>500 – 550 IR | 880 | 2 |
| Sup. S2A | 0.41 | 1 | 20x/0.8 | META:<br>373 – 383<br>437 – 458<br>512 – 533 | 760 | - |
| Sup. S2B | 0.82 | 1 | 20x/0.8 | META:<br>373 – 383<br>437 – 458<br>512 – 533 | 760 | 2 |
| Sup. S2C | 0.82 | 1 | 10x/0.45 | META:<br>373 – 383<br>437 – 458<br>512 – 533 | 760 | - |
| Sup. S3A - E | 0.82 | 1 | 20x/0.8 | META:<br>373 – 383<br>437 – 458<br>512 – 533 | 760 | 3 |

| | | | | | | |
|---|---|---|---|---|---|---|
| Sup. S4A | 0.21 | 1 | 20x/0.8 | 500 – 550 IR<br>575 – 615 IR | 760 | - |
| Sup. S4B | 0.21 | 1 | 20x/0.8 | 500 – 550 IR<br>575 – 615 IR | 760 | - |
| Sup. 5A | 0.82 | 1 | 20x/0.8 | 390 – 465 IR<br>500 – 550 IR | 880 | 2 |
| Sup. 6B | 1.64 | 1 | 10x/0.45 | Band Pass:<br>435 – 485 IR<br>(390 – 465 IR<br>500 – 550 IR) | 760,<br>(880) | - |
| Supplementary Video 2 | 0.82 | 1 | 20x/0.8 | 435 – 485 IR<br>500 – 550 IR | 740 | |
| Supplementary Video 3 | 0.82 | 1 | 20x/0.8 | 390 – 465 IR<br>500 – 550 IR | 880 | |
| Supplementary Video 4 | 0.82 | 1 | 20x/0.8 | 390 – 465 IR<br>500 – 550 IR | 880 | |

**Table of observations**

| **Category of the observed event** | **Number of animals** |
|---|---|
| Tumor cells attached to each other | 4 |
| Tumor cells attached to the ovarian collagen | 7 |
| Tumor cells attached to the epithelial layer | 4 |
| Tumor cells extended on the collagen fibers | 4 |
| Tumor cells attached to the oviduct collagen | 3 |
| Tumor cells infiltrate to the inner side of the ovarian collagen | 4 |
| Formation of the tumor stroma outside the ovary | 7 |
| Invading tumor stroma | 4 |

**Supplementary Technical drawings**
