# Supplementary material for: A Novel Intravital Imaging Window for Longitudinal Microscopy of the Mouse Ovary
Source: Sci Rep. 2015 Jul 24;5:12446. doi: 10.1038/srep12446 (PMC4513547; doi:10.1038/srep12446)
Supplement: Supplementary Information [file srep12446-s7.pdf]

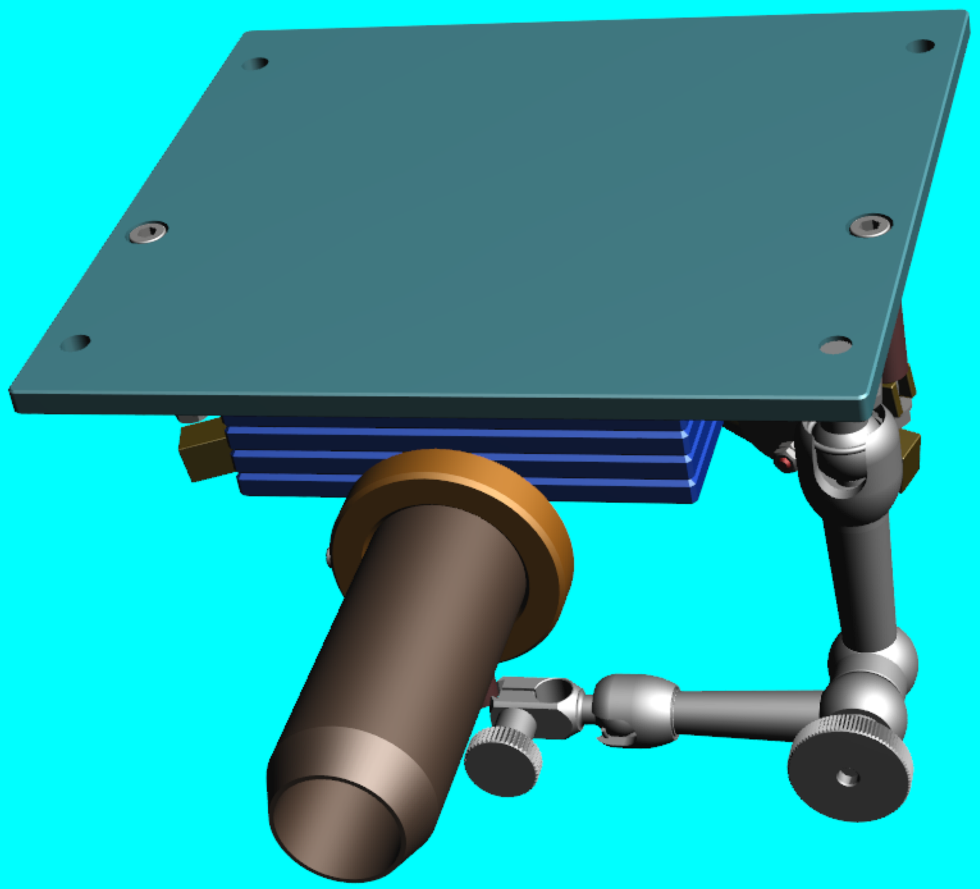

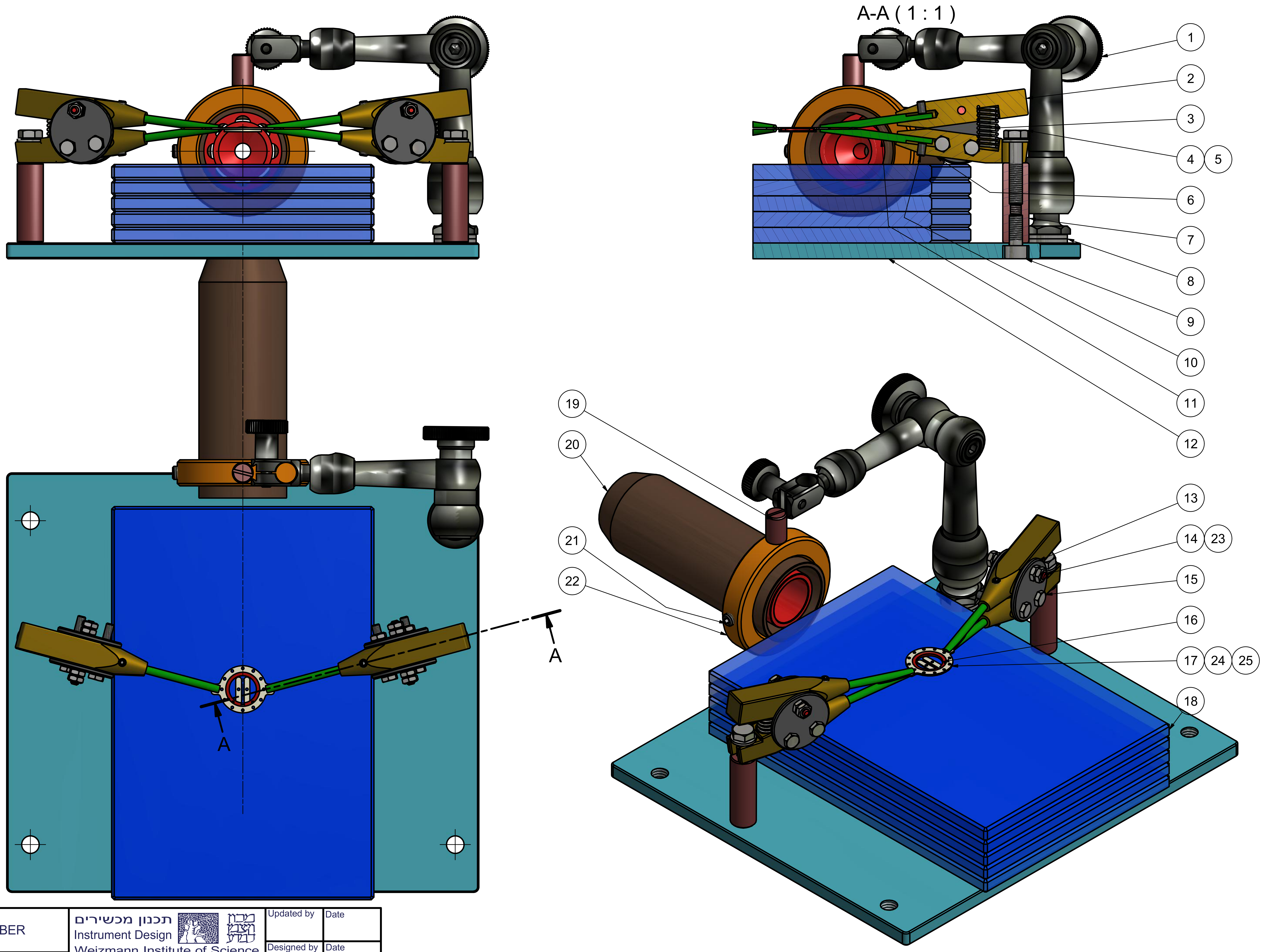

OVARIAN CHAMBER	תכנון מכשירים<br>Instrument Design<br>Weizmann Institute of Science<br>www.weizmann.ac.il/RSD/design	Updated by	Date
Ordered By<br>המחלקה לבקרה ביולוגית		Designed by<br>Lilia	Date<br>02/12/2012
Project Part Part Name<br>4777.00- Assembly1	Material		Quantity

ITEM	PART NUMBER	QTY	MATERIAL	DESCRIPTION	COMMENTS
25	Chamber3	1	Titanium		
24	Chamber2	1	Titanium		
23	Hexagon nut - M3	12	Stainless Steel		
22	Gas Conector Holder	1	Delrin, White		
21	Hexagon socket set screw - M5 x 10	1	Stainless Steel		
20	Gas Conector	1			
19	Pin	1	Delrin, White		
18	Spacer	5	Polycarbonate, Clear		
17	Chamber	1	Titanium		
16	Ring	1	Delrin, White		
15	Hexagon head bolt - M3 x 20	4	Stainless Steel		
14	Axis	2	Stainless Steel 303		
13	Disc	4	Stainless Steel 303		
12	Plate	1	Aluminum 6061		
11	Plier	4	Stainless Steel 316		
10	Hexagon socket set screw - M3 x 5	4	Stainless Steel		
9	Hexagon Socket Head Cap Screw - M5 x 12	2	Stainless Steel		
8	Plain washer - 8	2	Stainless Steel		
7	Post	2	Stainless Steel 303		
6	Plier Holder	2	Stainless Steel 303		
5	Plain washer - 5	2	Stainless Steel		
4	Hexagon head bolt - M5 x 25	2	Stainless Steel		
3	Spring	2	Stainless Steel		
2	Hand	2	Stainless Steel 303		
1	Noga	1			NOGA FLEX NF1010 3-303488

Parts List

OVARIAN CHAMBER	תכנון מכשירים<br>Instrument Design<br>Weizmann Institute of Science<br>www.weizmann.ac.il/RSD/design	Updated by	Date
Ordered By<br>המחלקה לבקרה ביולוגית		Designed by<br>Lilia	Date<br>02/12/2012
Project Part Part Name<br>4777.00- Assembly1	Material		Quantity

A-A (2 : 1)

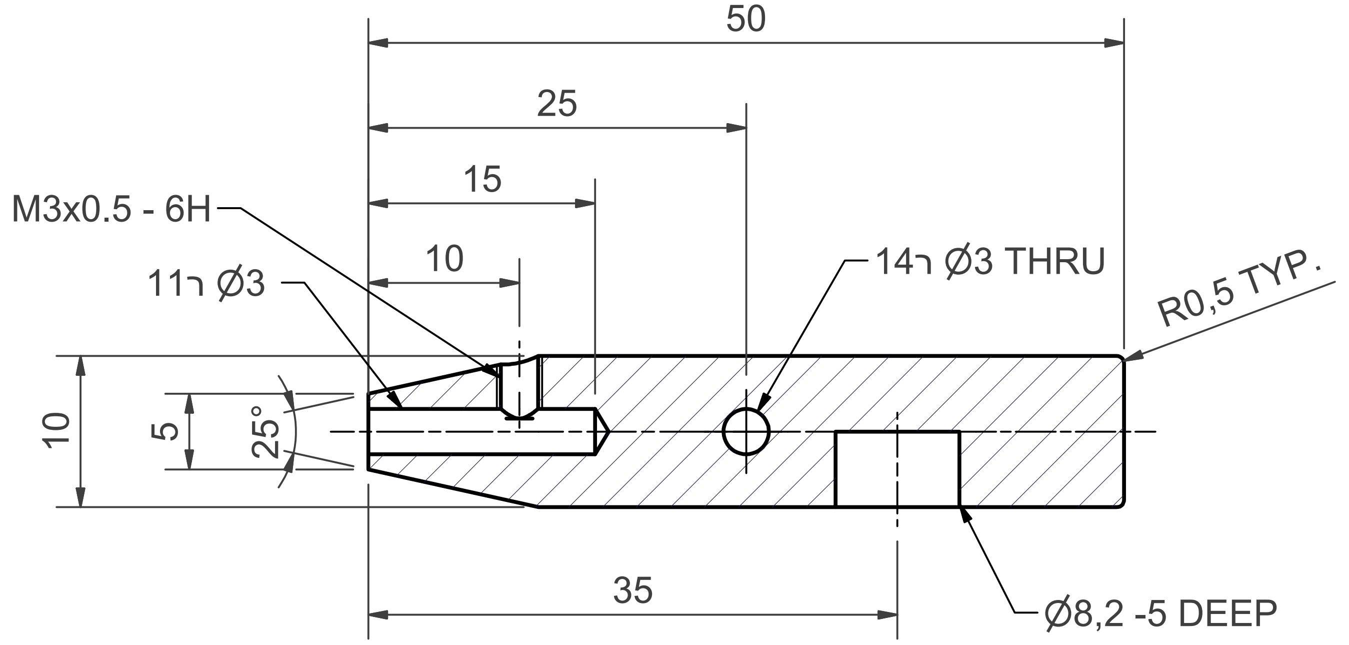

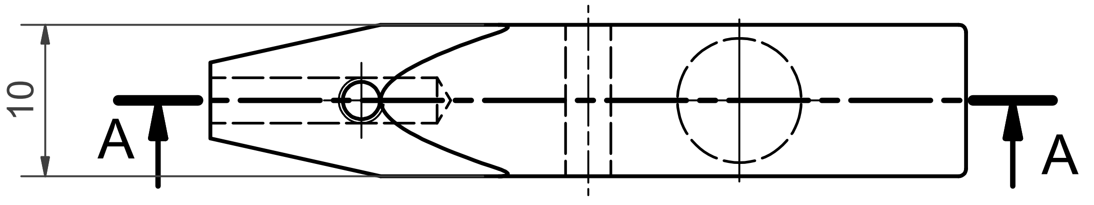

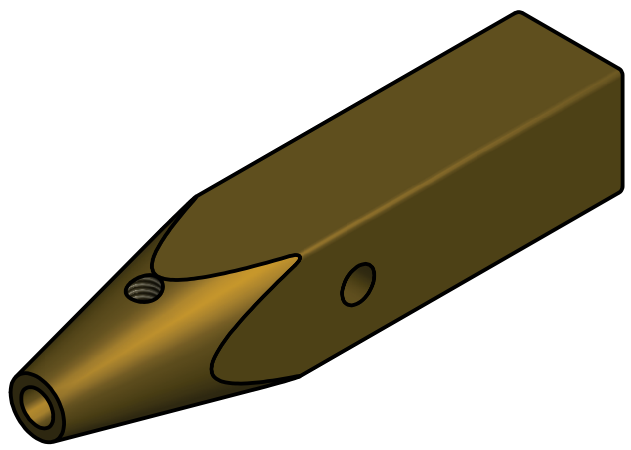

OVARIAN CHAMBER	תכנון מכשירים<br>Instrument Design<br>Weizmann Institute of Science<br>www.weizmann.ac.il/RSD/design	Updated by	Date
Ordered By<br>המחלקה לבקרה ביולוגית		Designed by<br>Lilia	Date<br>02/12/2012
Project Part Part Name<br>4777.00- 02 Hand	Material<br>Stainless Steel 303		Quantity

A-A (2 : 1)

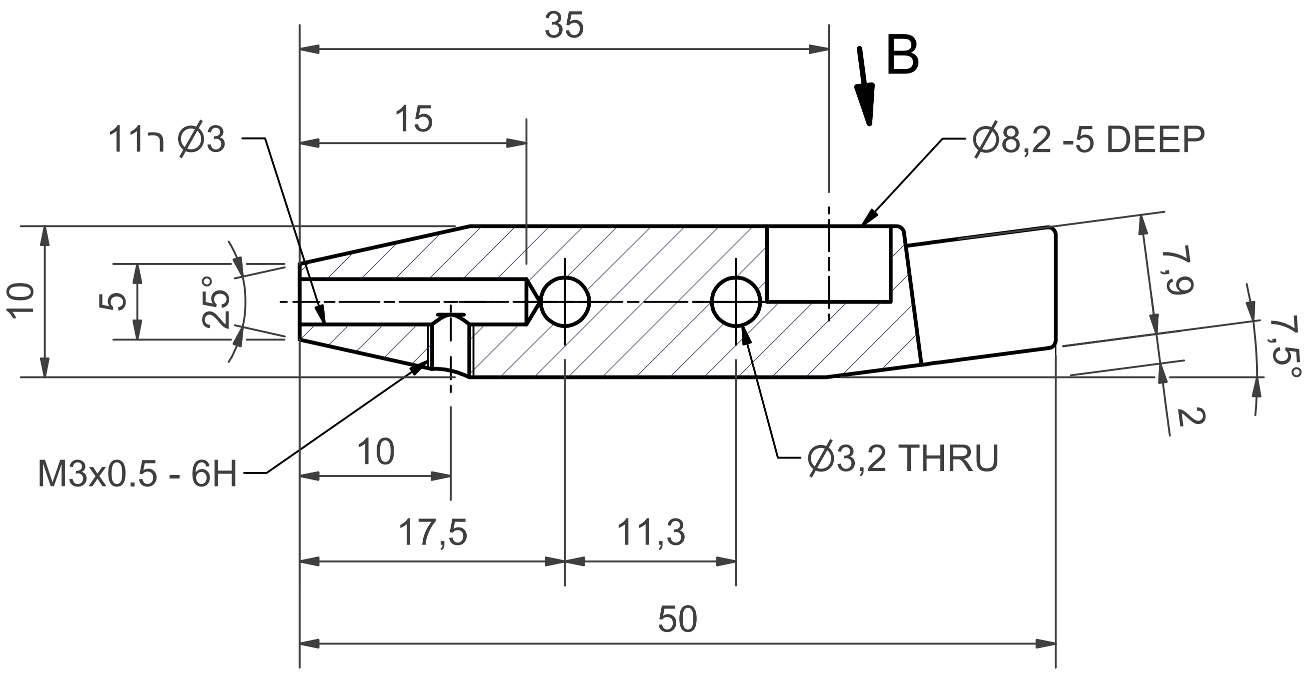

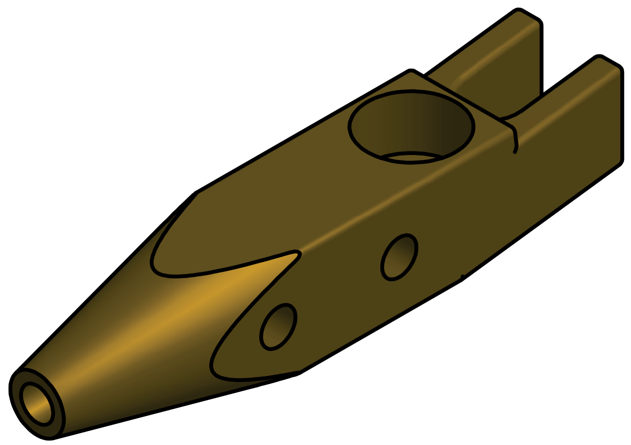

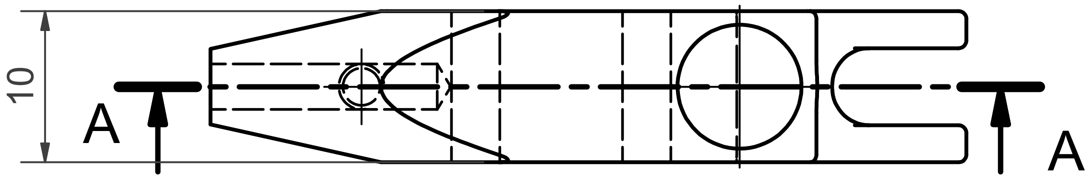

B (2 : 1)

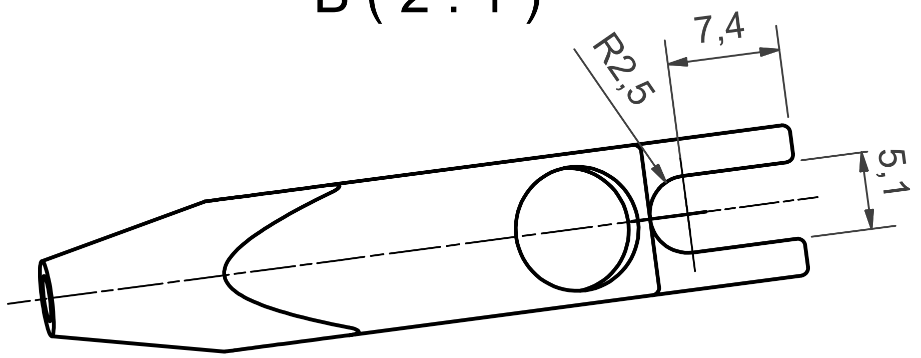

OVARIAN CHAMBER	מכון ויצמן למדע<br>פירישכמ וננכת<br>Instrument Design<br>Weizmann Institute of Science<br>www.weizmann.ac.il/RSD/design	Updated by	Date
Ordered By<br>המחלקה לבקרה ביולוגית		Designed by<br>Lilia	Date<br>02/12/2012
Project Part Part Name<br>4777.00- 06 Plier Holder	Material<br>Stainless Steel 303		Quantity

A-A (4 : 1)

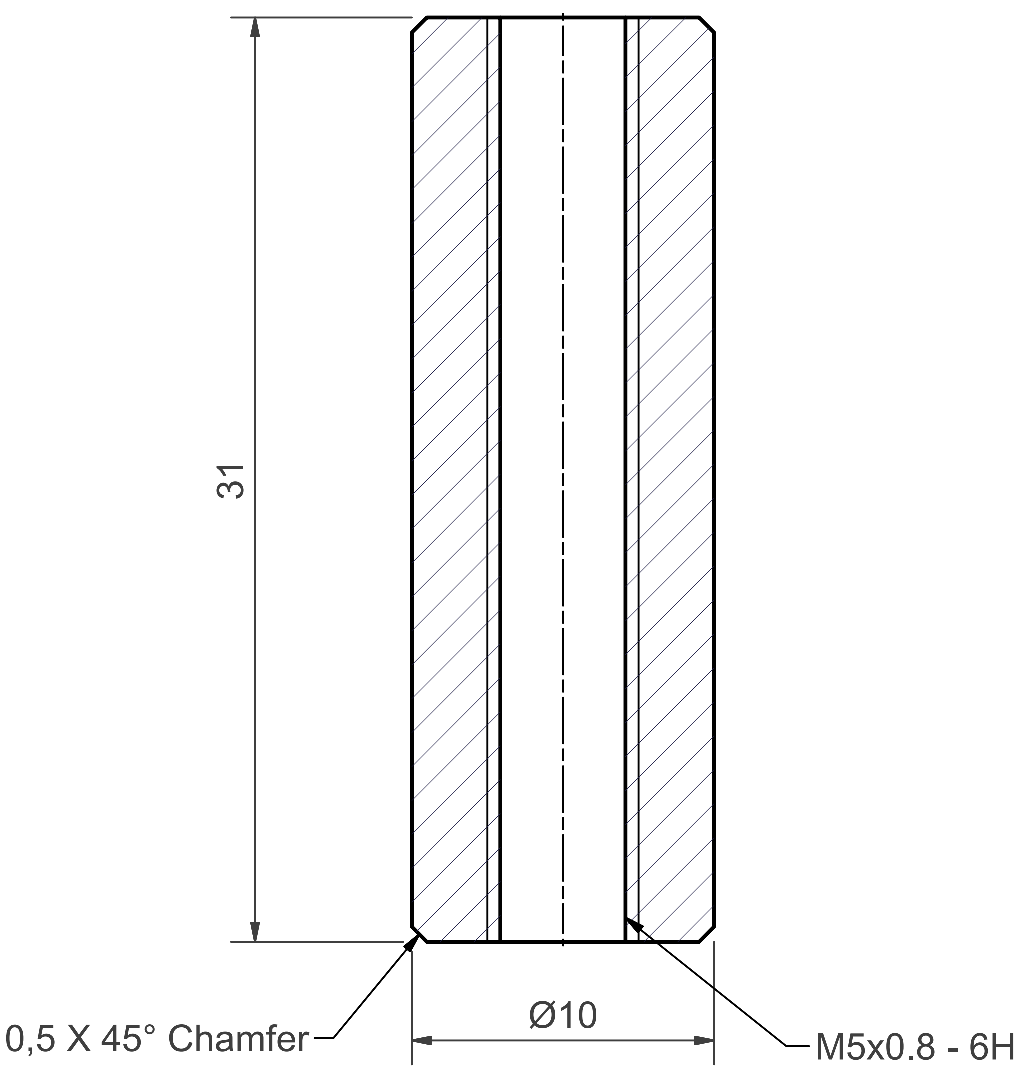

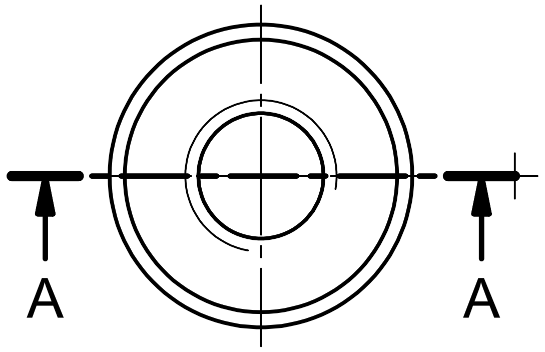

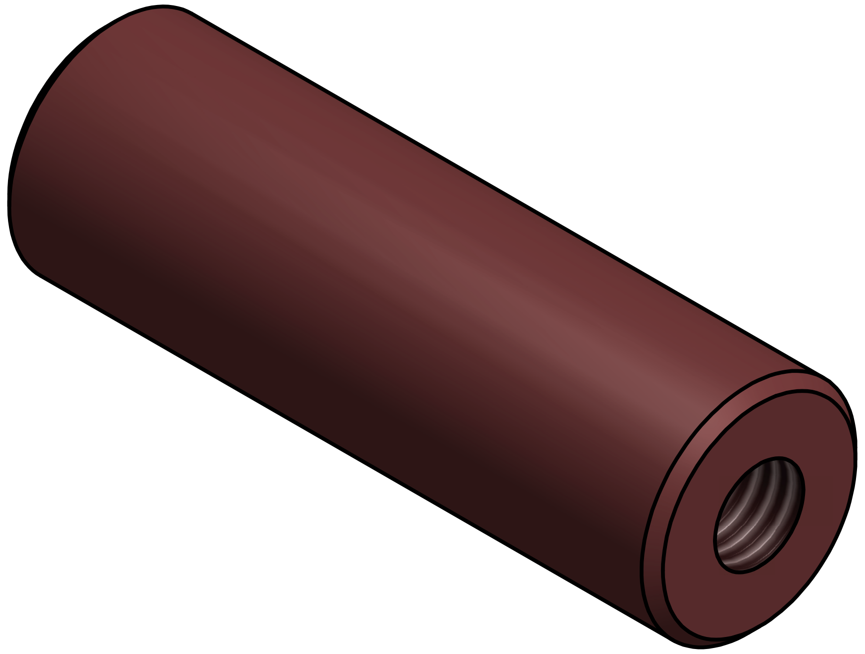

OVARIAN CHAMBER	תכנון ומכשירים<br>Instrument Design<br>Weizmann Institute of Science<br>www.weizmann.ac.il/RSD/design	Updated by	Date
Ordered By<br>המחלקה לבקרה ביולוגית		Designed by<br>Lilia	Date<br>02/12/2012
Project Part Part Name<br>4777.00- 07 Post	Material<br>Stainless Steel 303		Quantity

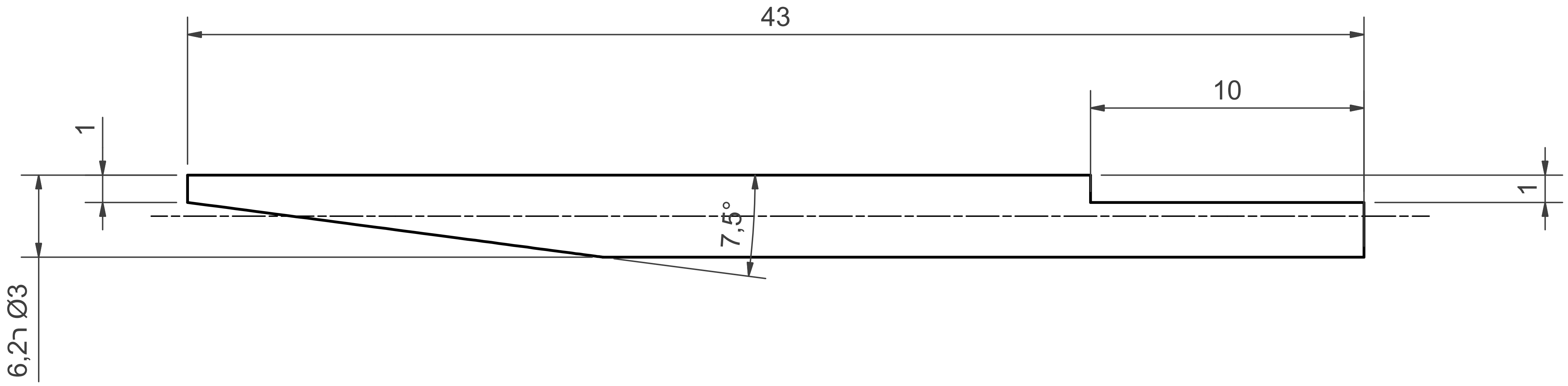

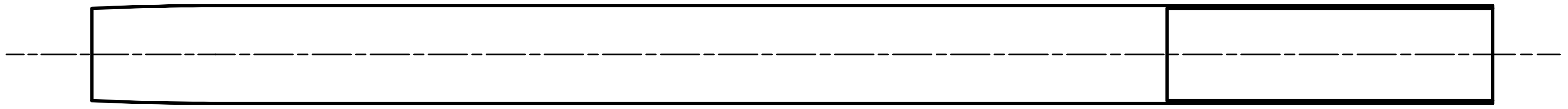

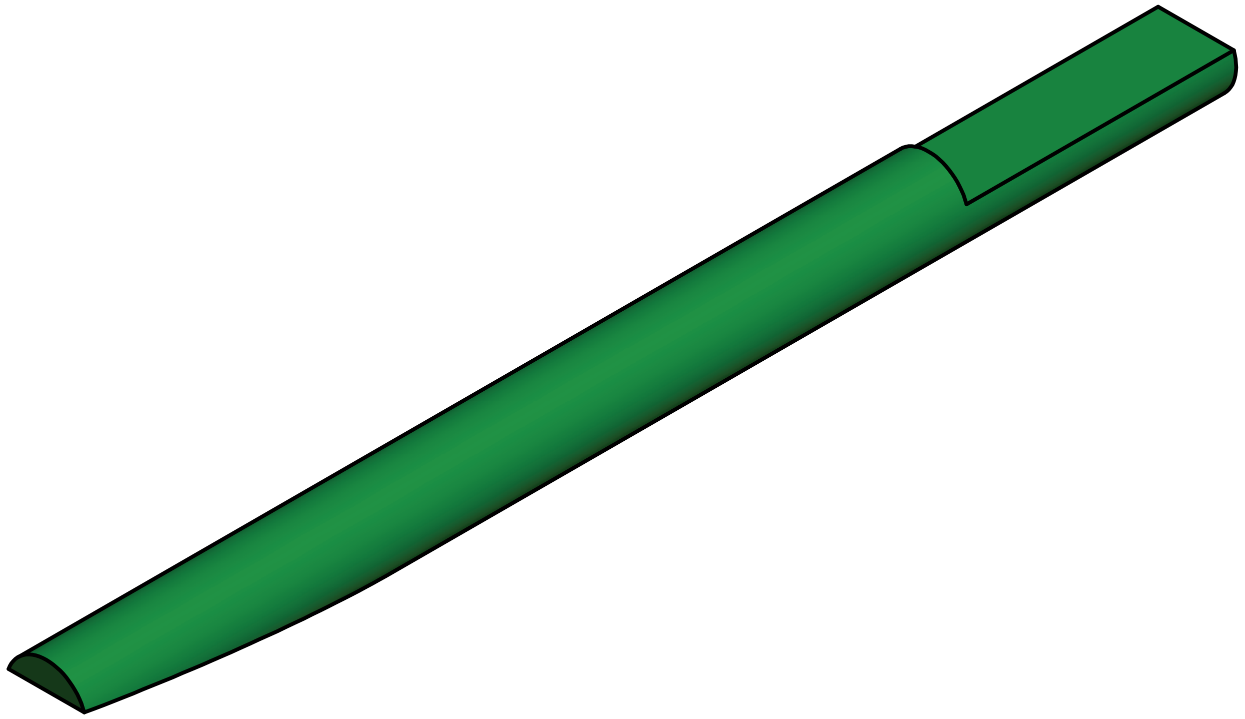

OVARIAN CHAMBER	תכנון מכשירים<br>Instrument Design<br>Weizmann Institute of Science<br>www.weizmann.ac.il/RSD/design	Updated by	Date
Ordered By<br>המחלקה לבקרה ביולוגית		Designed by<br>Lilia	Date<br>02/12/2012
Project 4777.00- Part 11 Part Name Plier	Material<br>Stainless Steel 316		Quantity

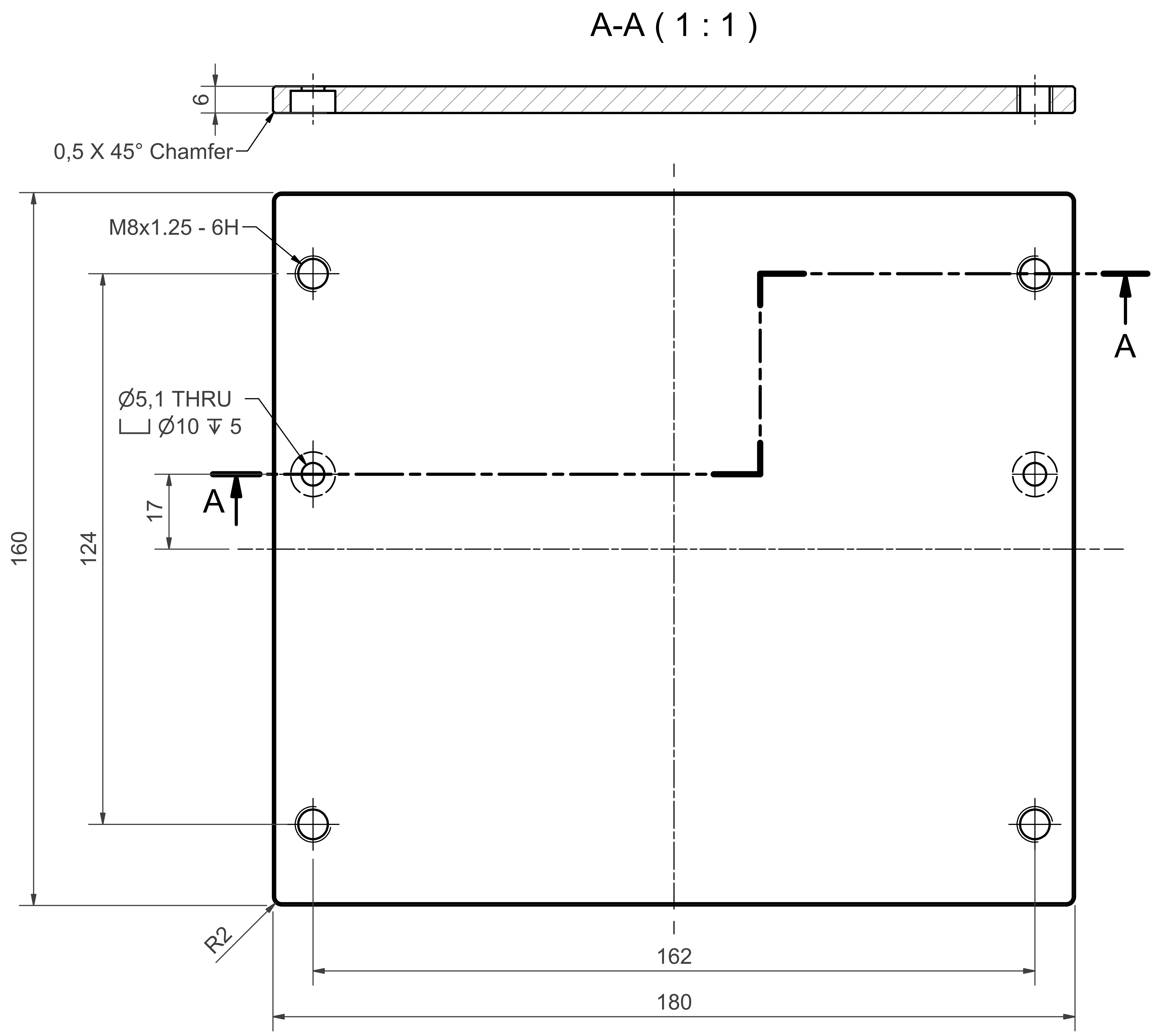

אנודייז שחור מט

OVARIAN CHAMBER	מכון ויצמן למדע<br>מכשירים ונכת<br>Instrument Design<br>Weizmann Institute of Science<br>www.weizmann.ac.il/RSD/design	Updated by	Date
Ordered By<br>המחלקה לבקרה ביולוגית		Designed by<br>Lilia	Date<br>02/12/2012
Project Part Part Name<br>4777.00- 12 Plate	Material<br>Aluminum 6061		Quantity

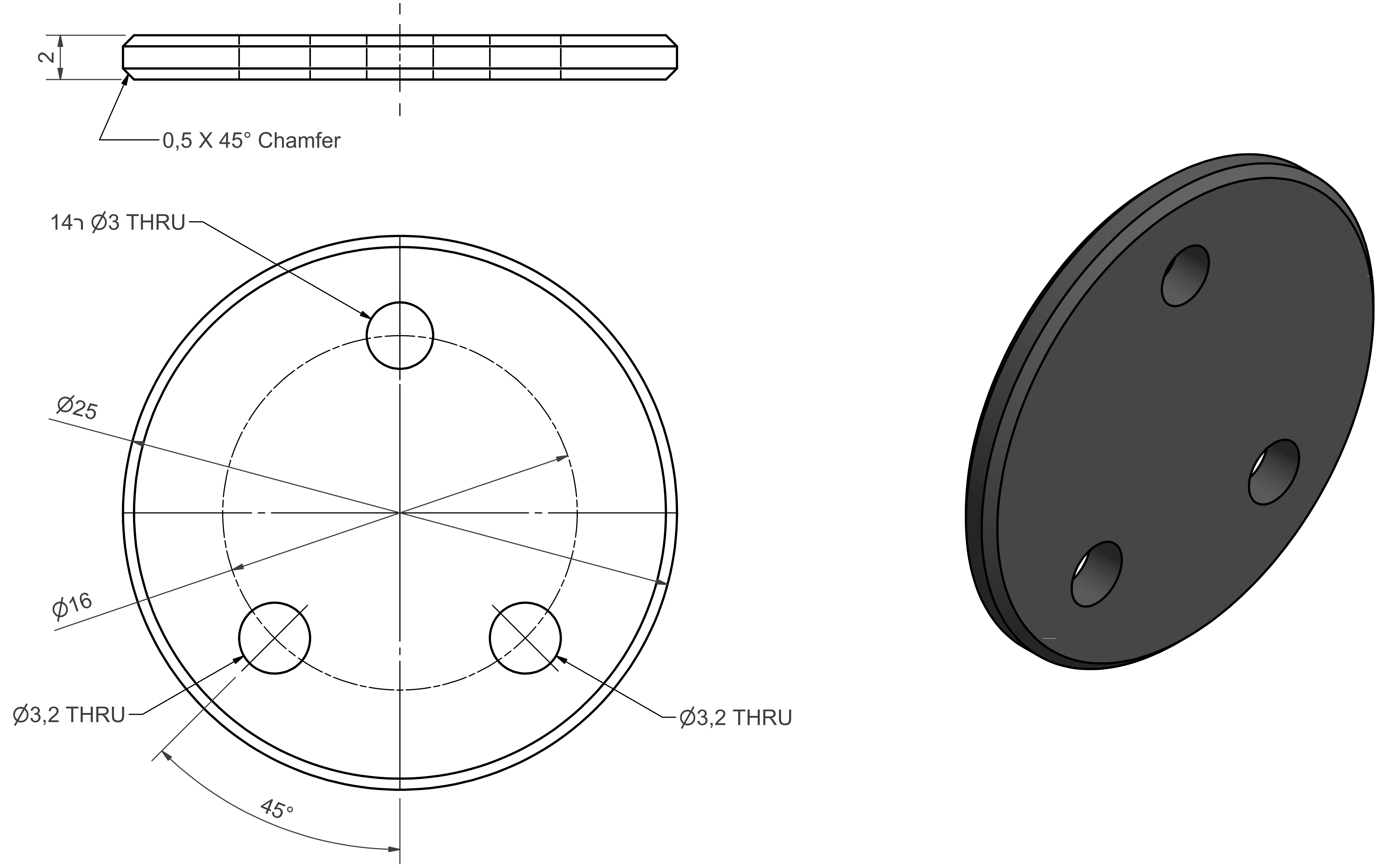

OVARIAN CHAMBER	תכנון מכשירים<br>Instrument Design<br>Weizmann Institute of Science<br>www.weizmann.ac.il/RSD/design	Updated by	Date
Ordered By<br>המחלקה לבקרה ביולוגית		Designed by<br>Lilia	Date<br>02/12/2012
Project Part Part Name<br>4777.00- 13 Disc	Material<br>Stainless Steel 303		Quantity

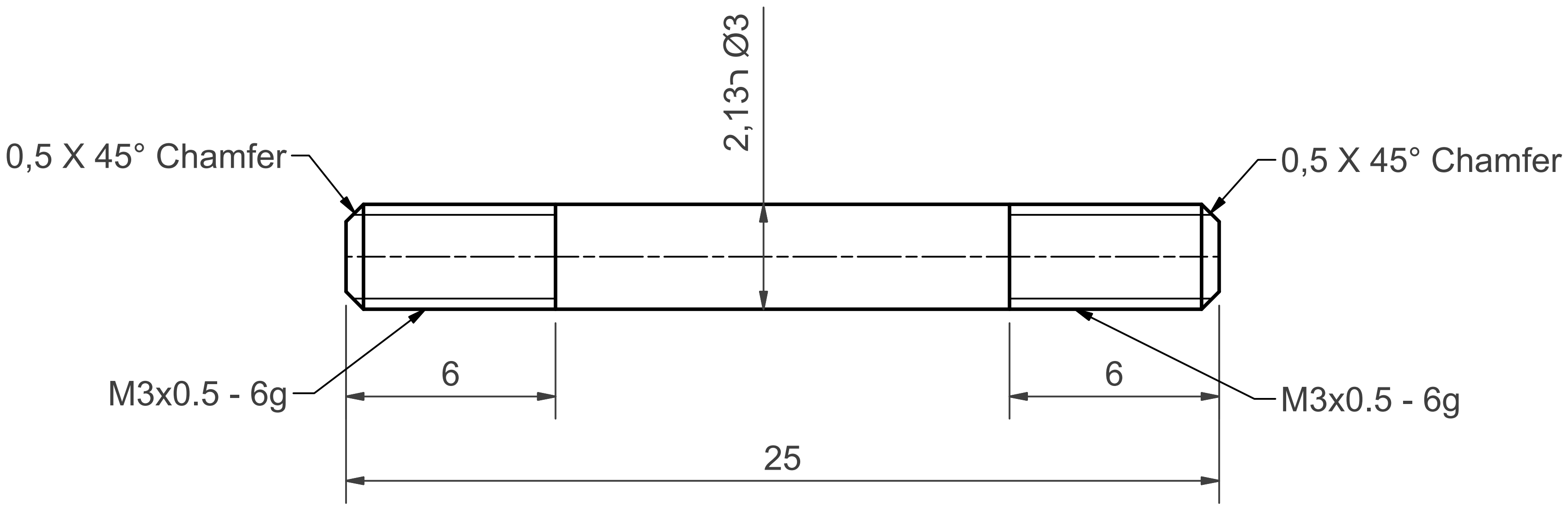

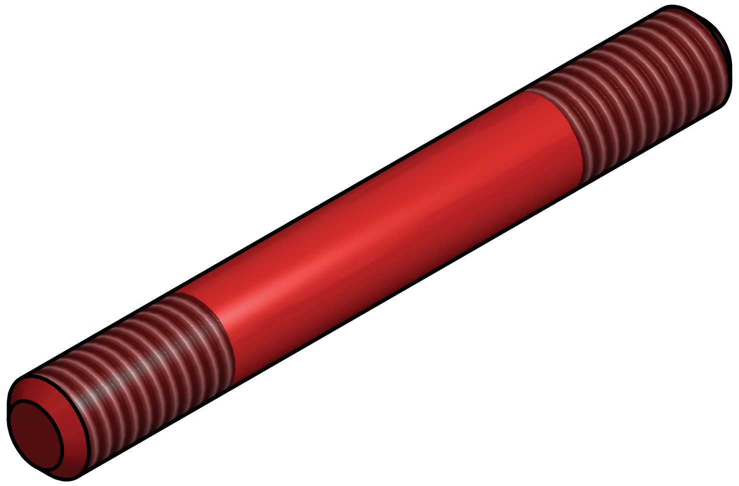

OVARIAN CHAMBER	מירישכם וננכת<br>Instrument Design<br>Weizmann Institute of Science<br>www.weizmann.ac.il/RSD/design	Updated by	Date
Ordered By<br>המחלקה לבקרה ביולוגית		Designed by<br>Lilia	Date<br>02/12/2012
Project 4777.00- Part 14 Part Name Axis	Material<br>Stainless Steel 303		Quantity

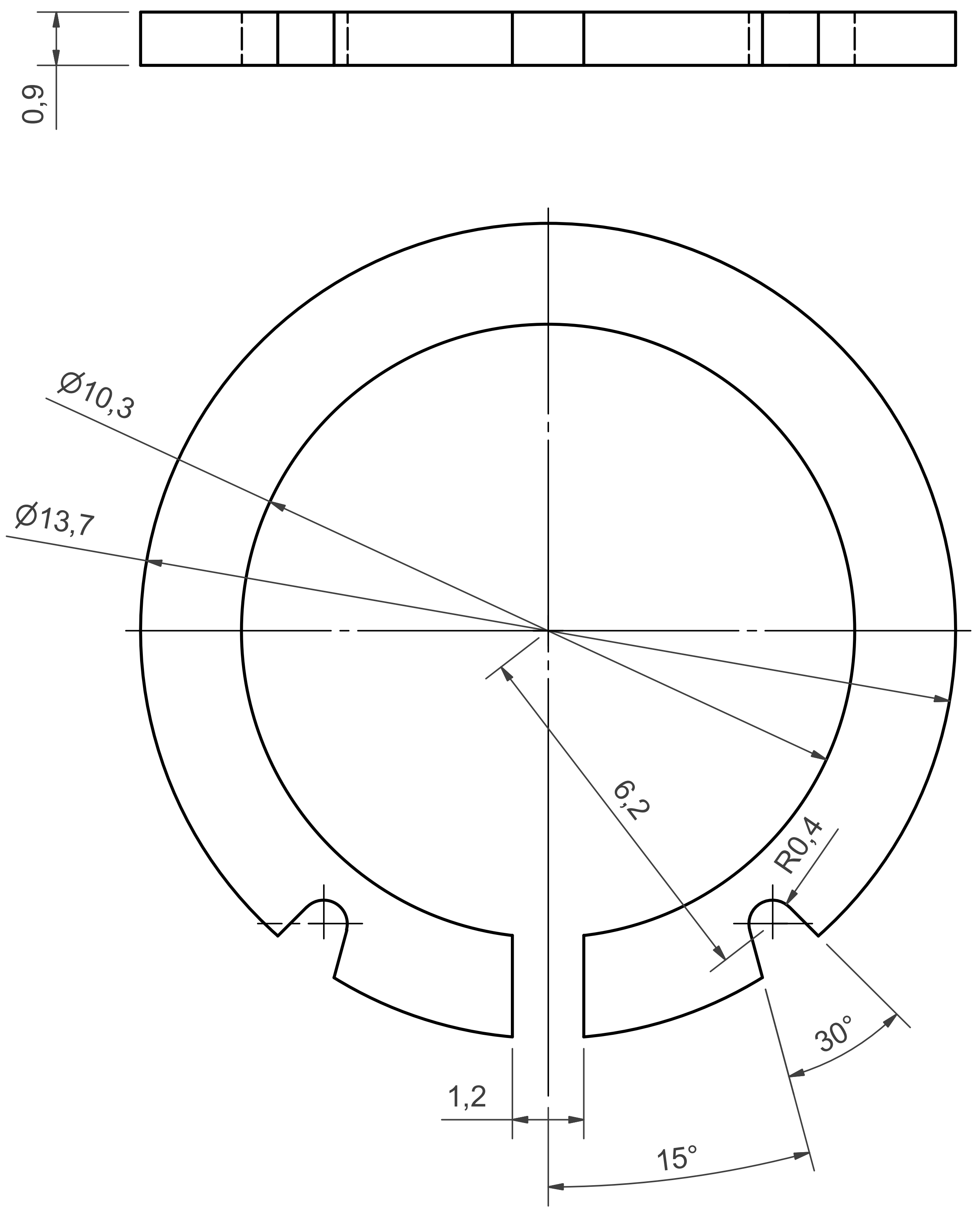

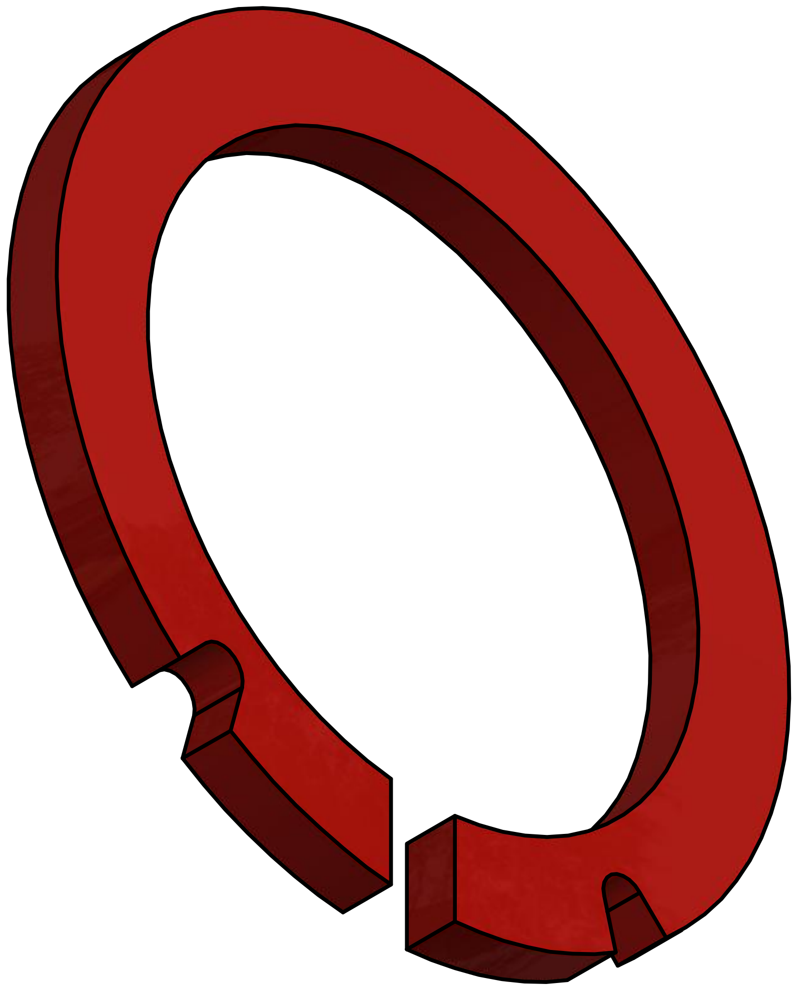

OVARIAN CHAMBER

Ordered By
המחלקה לבקרה ביולוגית

תכנון מכשירים
Instrument Design
Weizmann Institute of Science
www.weizmann.ac.il/RSD/design

Updated by	Date
Designed by<br>Lilia	Date<br>02/12/2012

Project	Part	Part Name	Material	Quantity
4777.00-	16	Ring	Delrin, White	

A-A (5 : 1)

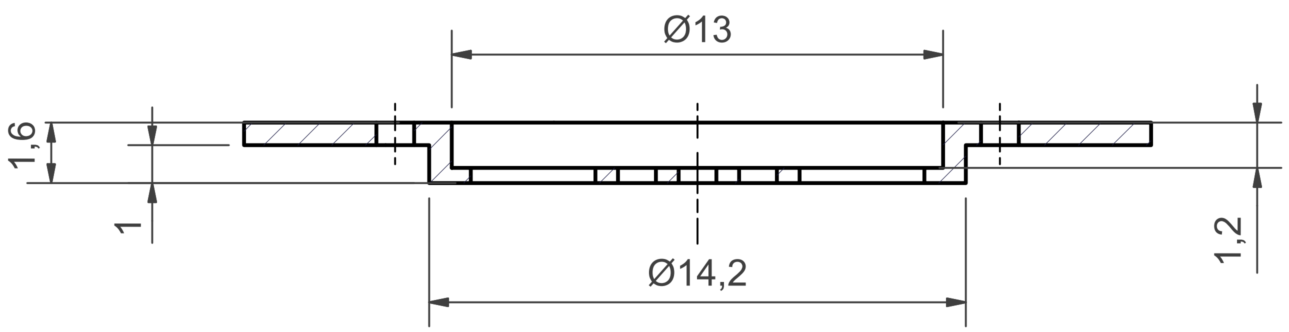

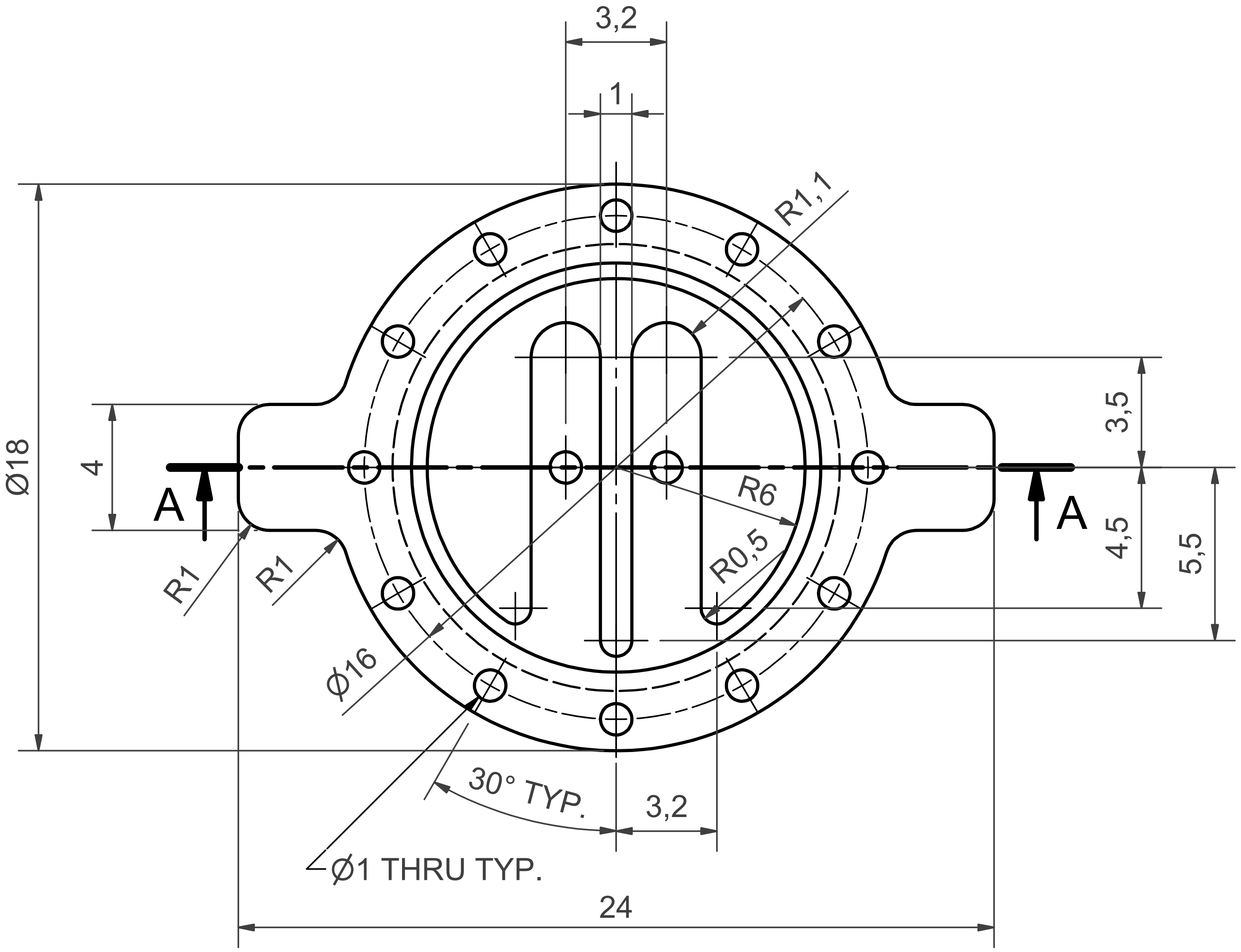

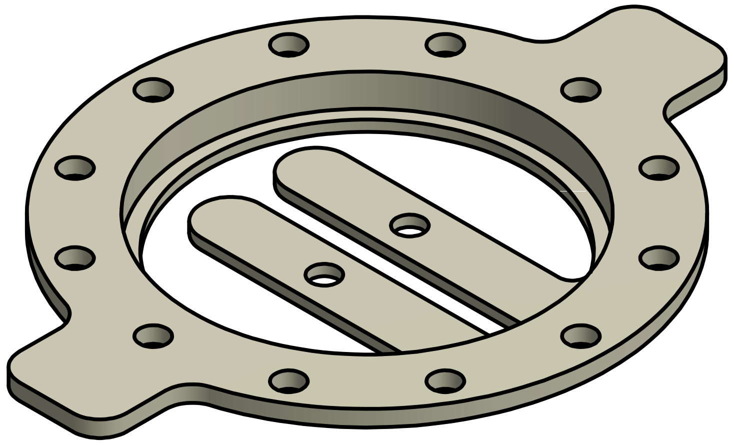

OVARIAN CHAMBER	מכון ויצמן למדע<br>מחלקת מכשירים<br>Instrument Design<br>Weizmann Institute of Science<br>www.weizmann.ac.il/RSD/design	Updated by	Date
Ordered By<br>המחלקה לבקרה ביולוגית		Designed by<br>Lilia	Date<br>02/12/2012
Project Part Part Name<br>4777.00- 17 Chamber	Material<br>Titanium		Quantity

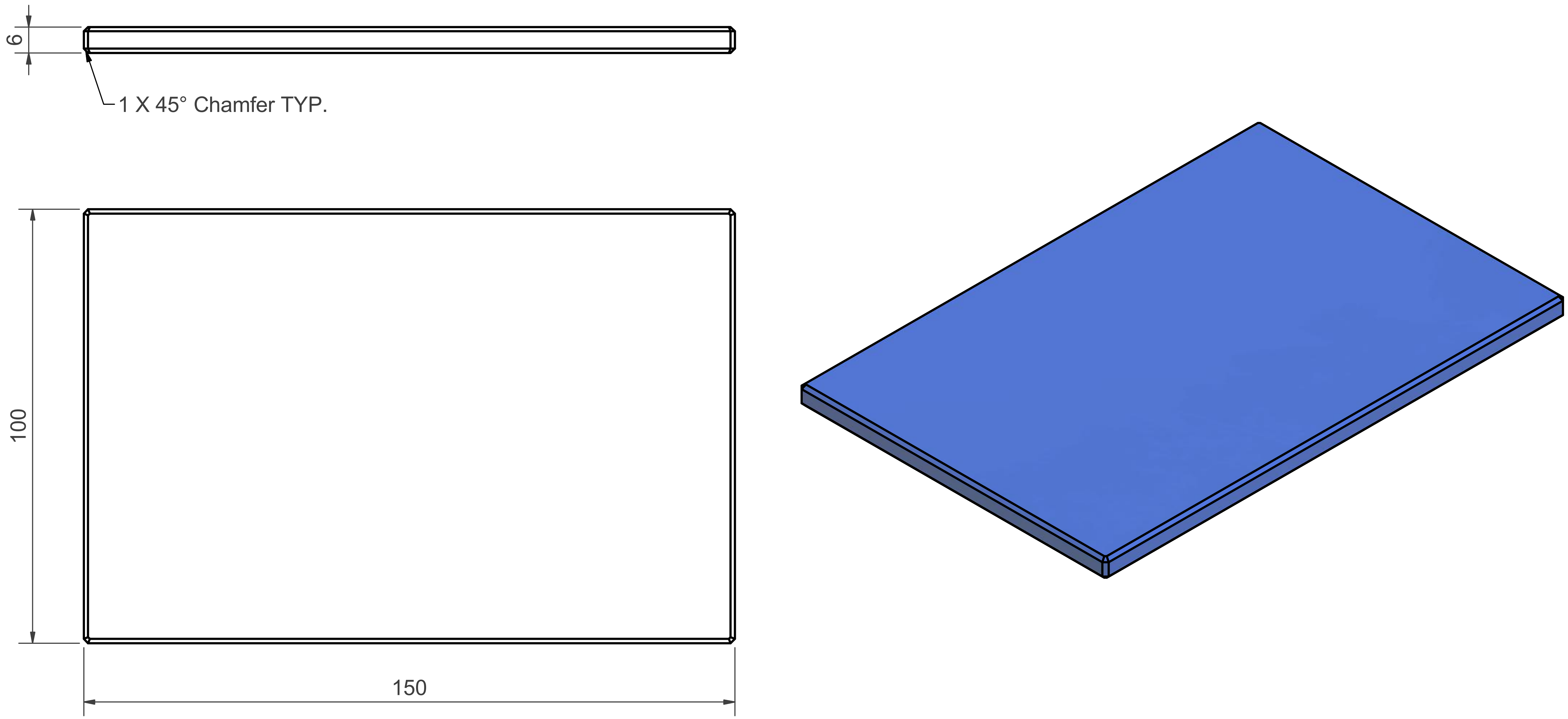

OVARIAN CHAMBER	תכנון מכשירים<br>Instrument Design<br>Weizmann Institute of Science<br>www.weizmann.ac.il/RSD/design	Updated by	Date
Ordered By<br>המחלקה לבקרה ביולוגית		Designed by<br>Lilia	Date<br>02/12/2012
Project 4777.00- / Part 18 / Part Name Spacer	Material<br>Polycarbonate, Clear		Quantity

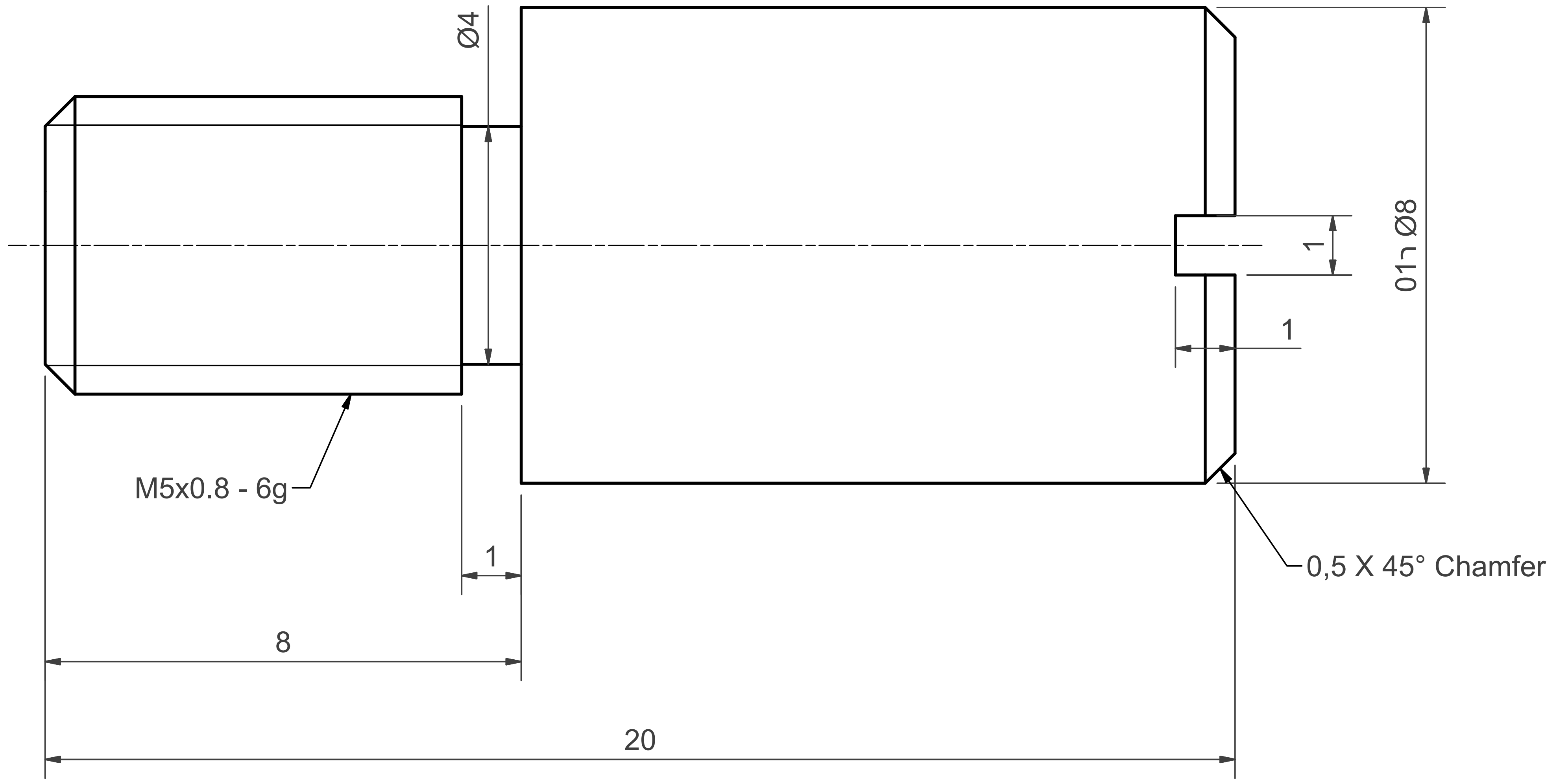

OVARIAN CHAMBER	תכנון ומכשירים<br>Instrument Design<br>Weizmann Institute of Science<br>www.weizmann.ac.il/RSD/design	Updated by	Date
Ordered By<br>המחלקה לבקרה ביולוגית		Designed by<br>Lilia	Date<br>02/12/2012
Project Part Part Name<br>4777.00- 19 Pin	Material<br>Delrin, White		Quantity

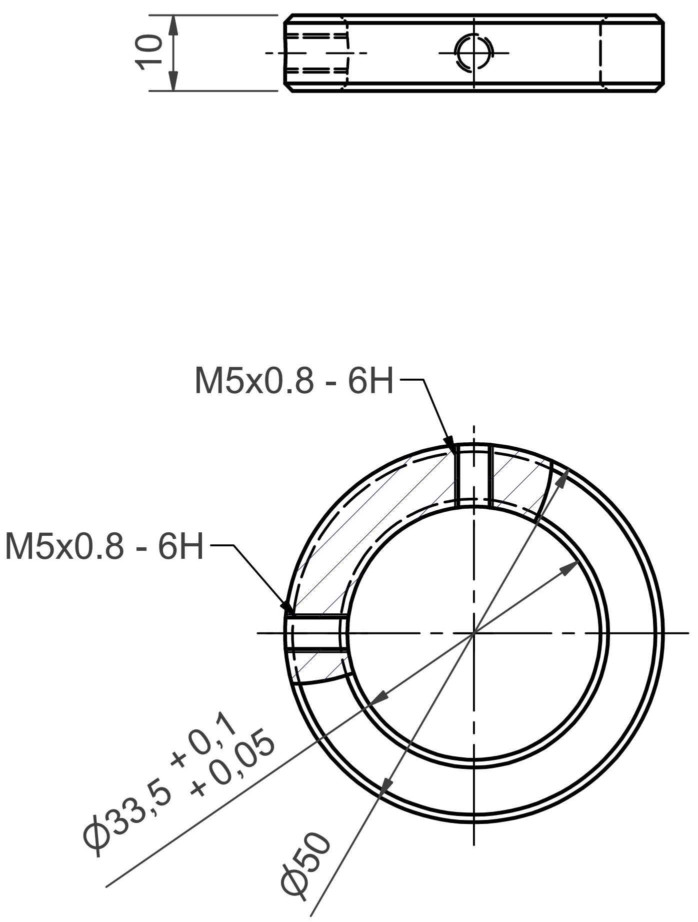

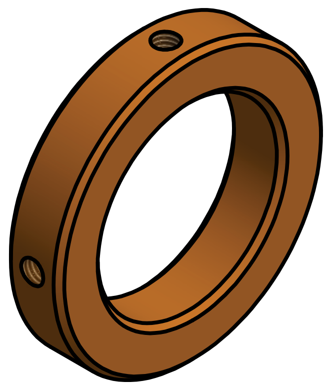

OVARIAN CHAMBER	מכון ויצמן למדע<br>תכנון מכשירים<br>Instrument Design<br>Weizmann Institute of Science<br>www.weizmann.ac.il/RSD/design	Updated by	Date
Ordered By<br>המחלקה לבקרה ביולוגית		Designed by<br>Lilia	Date<br>02/12/2012
Project 4777.00- Part 22 Part Name Gas Connector Holder	Material<br>Delrin, White		Quantity

A-A (5 : 1)

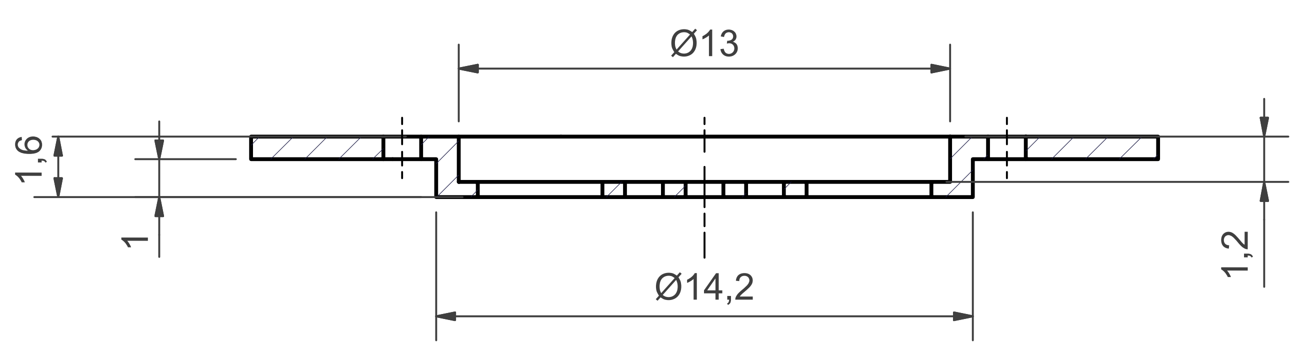

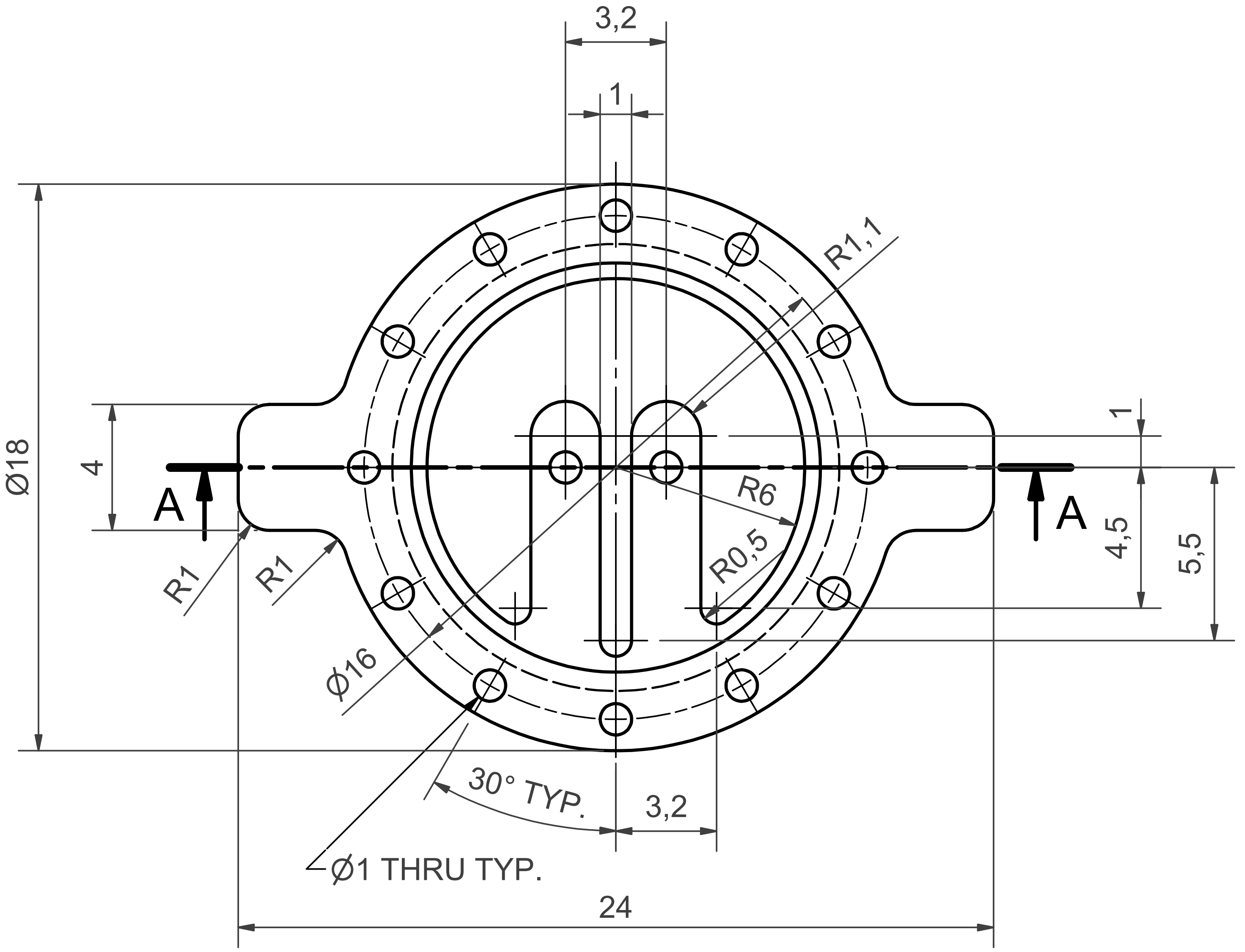

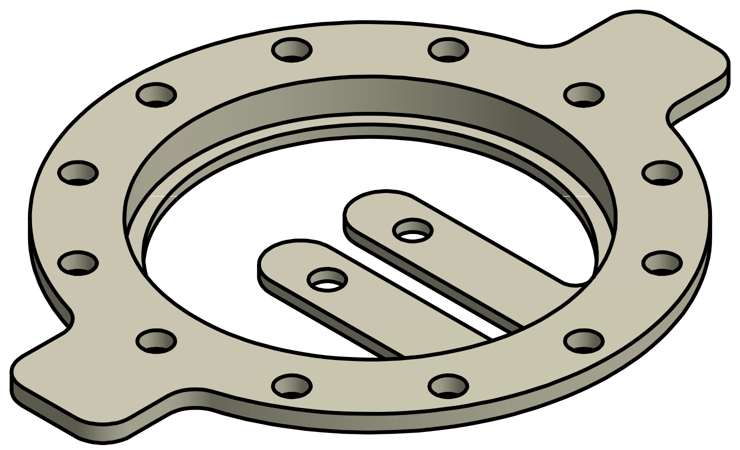

OVARIAN CHAMBER	מכון ויצמן למדע<br>מחלקת מכשור ופיתוח<br>Instrument Design<br>Weizmann Institute of Science<br>www.weizmann.ac.il/RSD/design	Updated by	Date
Ordered By<br>המחלקה לבקרה ביולוגית		Designed by<br>Lilia	Date<br>02/12/2012
Project Part Part Name<br>4777.00- 24 Chamber2	Material<br>Titanium		Quantity

A-A (5 : 1)

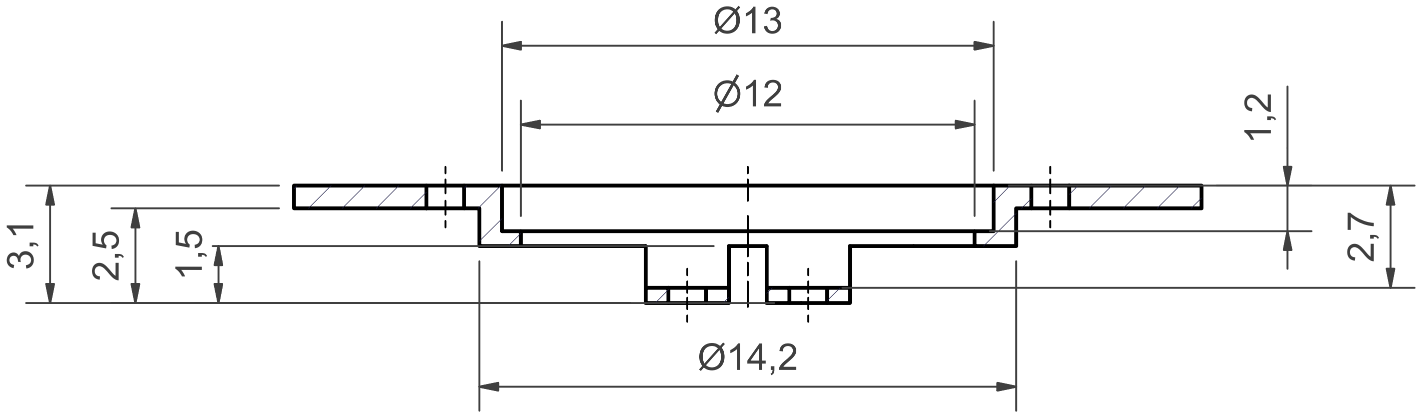

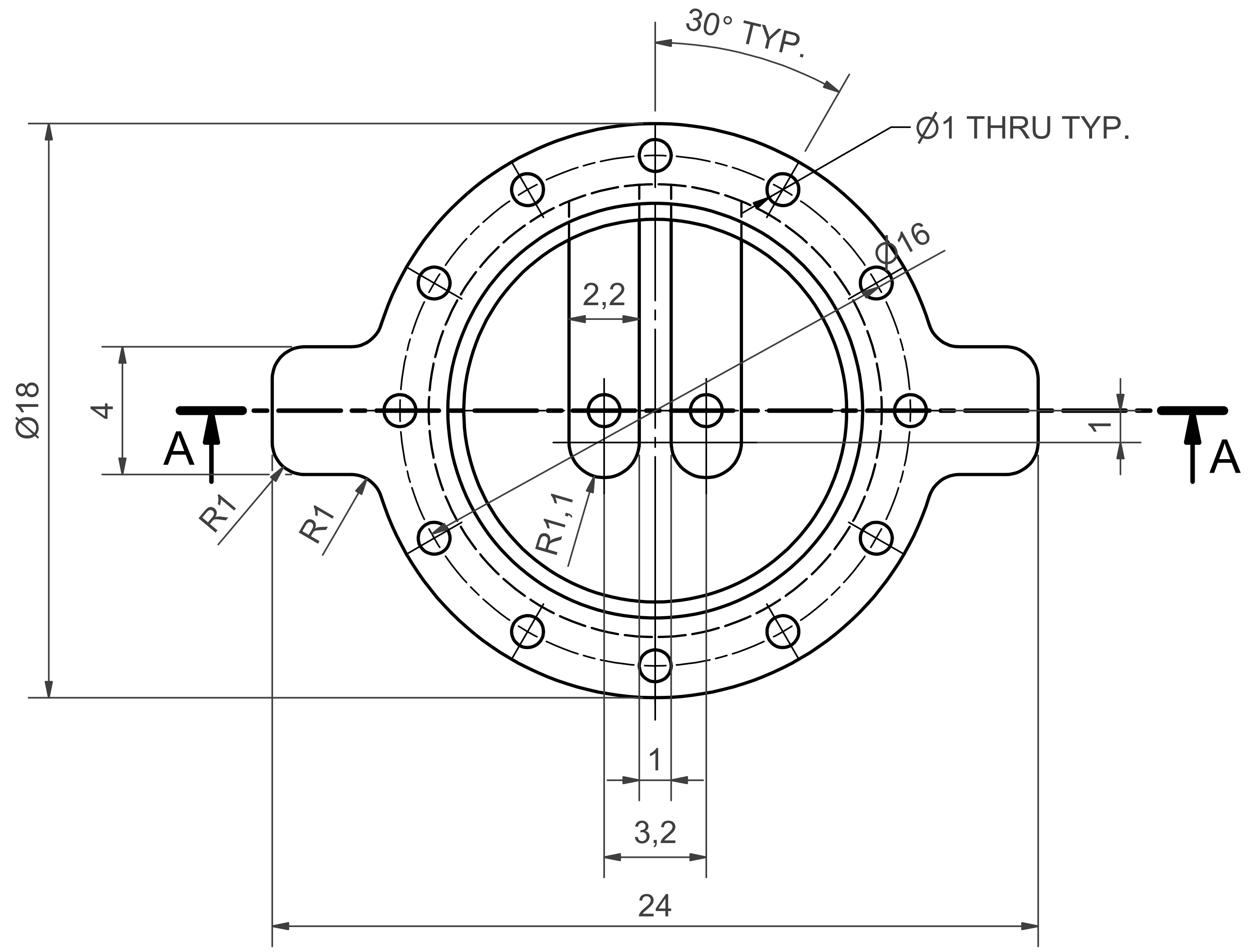

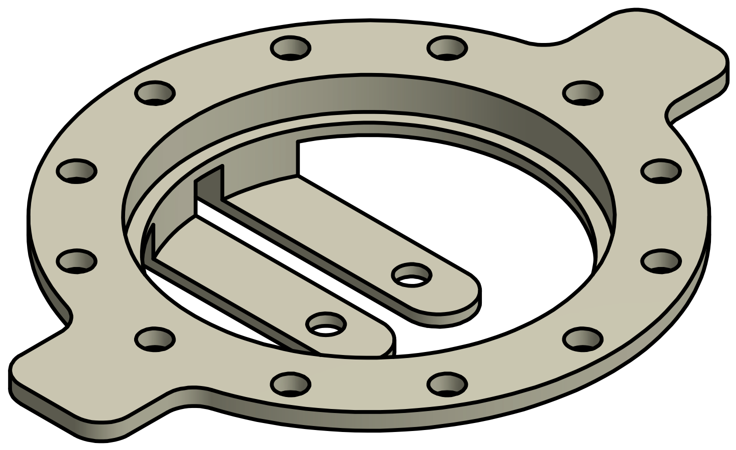

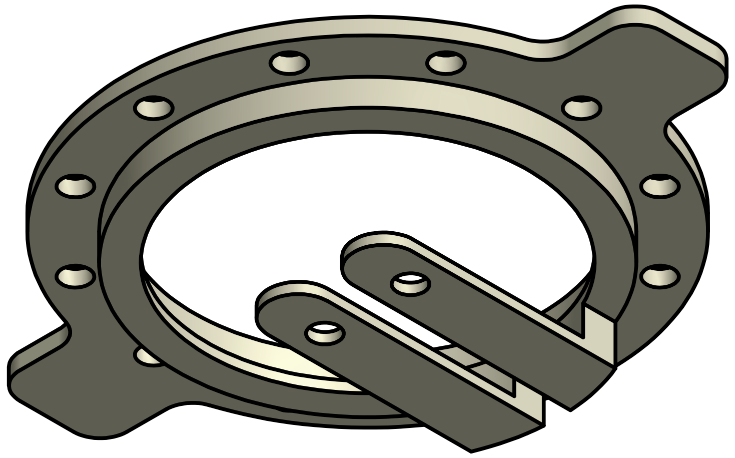

OVARIAN CHAMBER	מכון ויצמן למדע<br>מחלקת מכשירים<br>Instrument Design<br>Weizmann Institute of Science<br>www.weizmann.ac.il/RSD/design	Updated by	Date
Ordered By<br>המחלקה לבקרה ביולוגית		Designed by<br>Lilia	Date<br>02/12/2012
Project Part Part Name<br>4777.00- 25 Chamber3	Material<br>Titanium		Quantity

A-A (5 : 1)

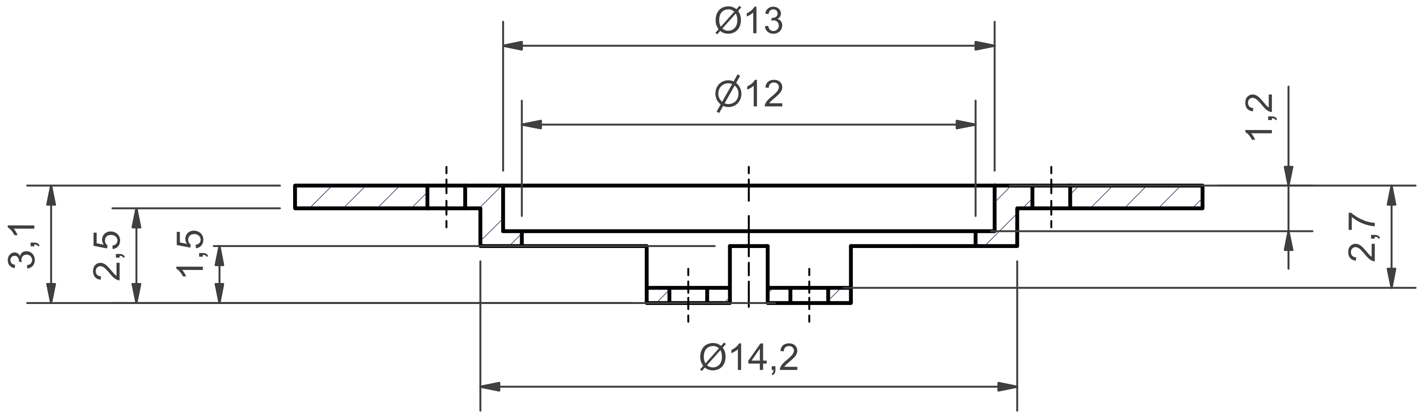

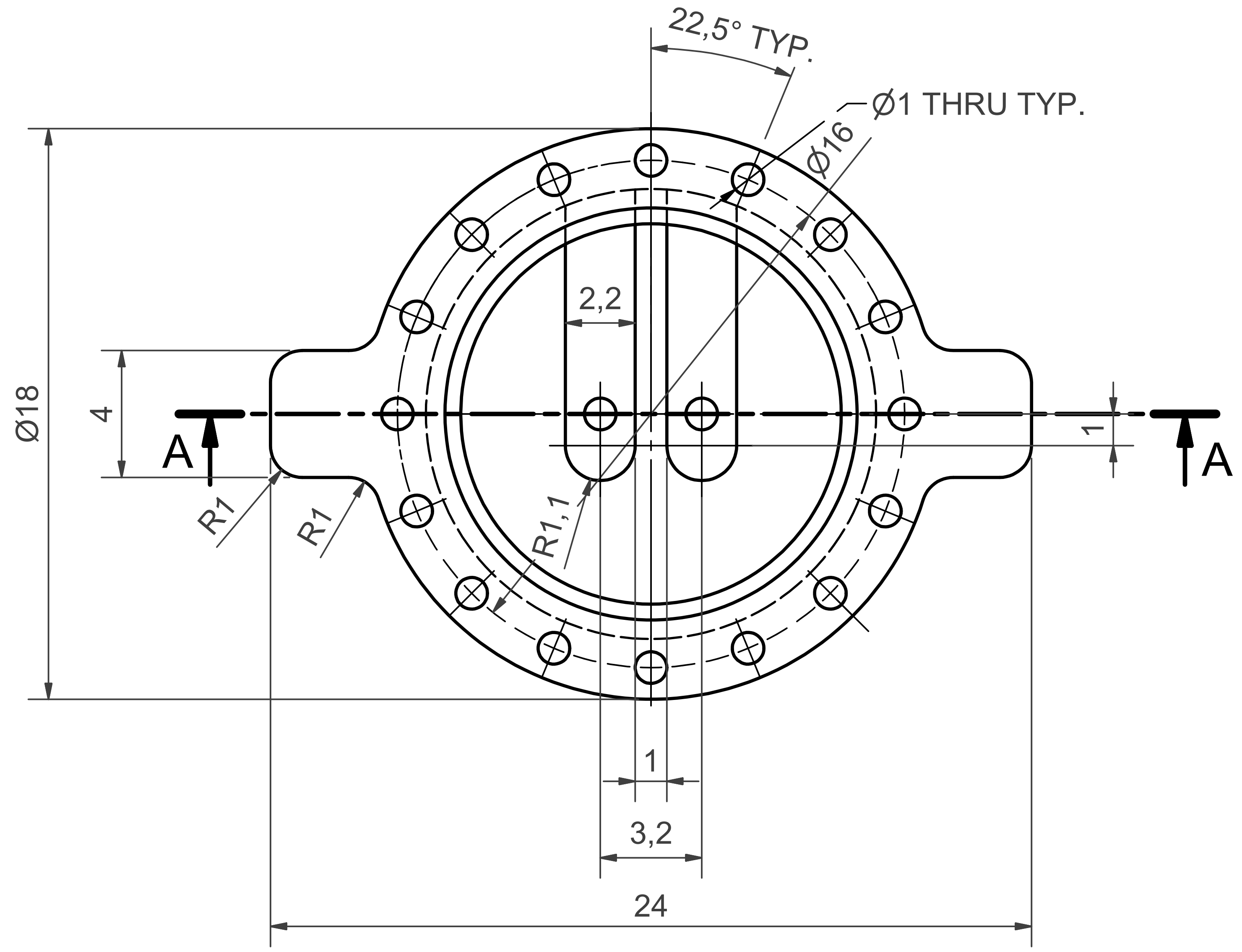

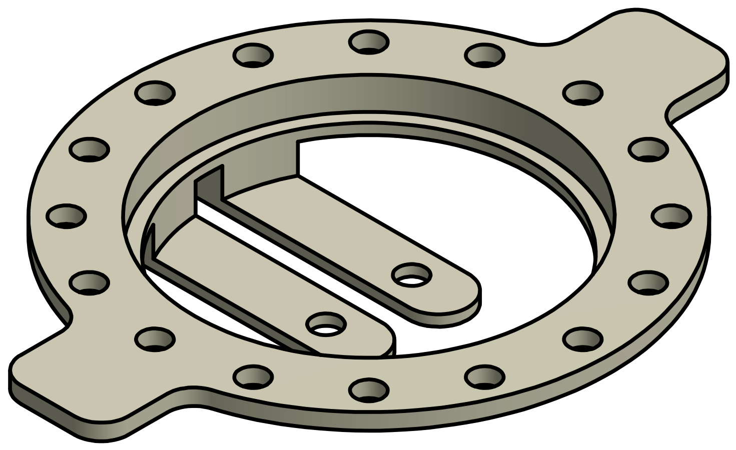

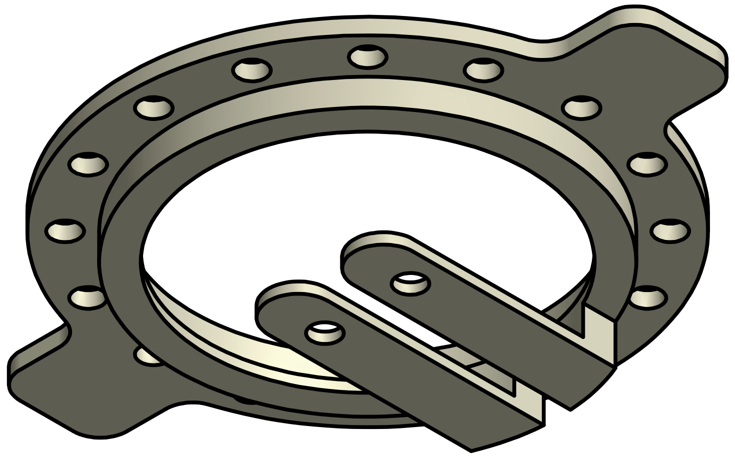

OVARIAN CHAMBER

Ordered By
המחלקה לבקרה ביולוגית

מחלקת מכשור ופיתוח
Instrument Design
Weizmann Institute of Science
www.weizmann.ac.il/RSD/design

Updated by | Date

Designed by: Lilia | Date: 02/12/2012

Project: 4777.00- | Part: 26 | Part Name: Chamber4

Material: Titanium

Quantity
